# Synaptic reorganization of synchronized neuronal networks with synaptic weight and structural plasticity

**DOI:** 10.1371/journal.pcbi.1012261

**Published:** 2024-07-09

**Authors:** Kanishk Chauhan, Alexander B. Neiman, Peter A. Tass

**Affiliations:** 1 Department of Physics and Astronomy, Ohio University, Athens, Ohio, United States of America; 2 Neuroscience Program, Ohio University, Athens, Ohio, United States of America; 3 Department of Neurosurgery, Stanford University, Stanford, California, United States of America; École Normale Supérieure, College de France, CNRS, FRANCE

## Abstract

Abnormally strong neural synchronization may impair brain function, as observed in several brain disorders. We computationally study how neuronal dynamics, synaptic weights, and network structure co-emerge, in particular, during (de)synchronization processes and how they are affected by external perturbation. To investigate the impact of different types of plasticity mechanisms, we combine a network of excitatory integrate-and-fire neurons with different synaptic weight and/or structural plasticity mechanisms: (i) only spike-timing-dependent plasticity (STDP), (ii) only homeostatic structural plasticity (hSP), i.e., without weight-dependent pruning and without STDP, (iii) a combination of STDP and hSP, i.e., without weight-dependent pruning, and (iv) a combination of STDP and structural plasticity (SP) that includes hSP and weight-dependent pruning. To accommodate the diverse time scales of neuronal firing, STDP, and SP, we introduce a simple stochastic SP model, enabling detailed numerical analyses. With tools from network theory, we reveal that structural reorganization may remarkably enhance the network’s level of synchrony. When weaker contacts are preferentially eliminated by weight-dependent pruning, synchrony is achieved with significantly sparser connections than in randomly structured networks in the STDP-only model. In particular, the strengthening of contacts from neurons with higher natural firing rates to those with lower rates and the weakening of contacts in the opposite direction, followed by selective removal of weak contacts, allows for strong synchrony with fewer connections. This activity-led network reorganization results in the emergence of degree-frequency, degree-degree correlations, and a mixture of degree assortativity. We compare the stimulation-induced desynchronization of synchronized states in the STDP-only model (i) with the desynchronization of models (iii) and (iv). The latter require stimuli of significantly higher intensity to achieve long-term desynchronization. These findings may inform future pre-clinical and clinical studies with invasive or non-invasive stimulus modalities aiming at inducing long-lasting relief of symptoms, e.g., in Parkinson’s disease.

## Author summary

Synaptic weight and structural plasticity of neuronal networks determine their behavior, and abnormalities therein may underlie disordered states. Studying how different plasticity mechanisms govern network dynamics, particularly during (de)synchronization processes, holds clinical importance concerning, e.g., Parkinson’s disease. The marked difference between the timescales at which neuronal spiking activity (milliseconds), synaptic weight modifications (minutes-hours), and structural changes (hours-days) occur in the brain makes plastic network models computationally expensive, which may limit the scope of studies. Here, we present a leaky integrate-and-fire (LIF) neuron network model with a standard spike-timing-dependent plasticity (STDP) rule for weight plasticity and a stochastic structural plasticity (SP) method. The model is computationally efficient, allowing for detailed numerical analyses of network dynamics and structure. Combining the model with tools from network science, we show that structural reorganization resulting from SP can optimize the network for synchronization, elevating the level of synchrony while concurrently reducing overall network connections. This leads to the emergence of structural correlations between the natural firing rates of neurons and the number of their pre- and post-synaptic partners. Additionally, we demonstrate that synchronized networks that evolved with SP can be more robust against desynchronization stimulation.

## Introduction

Neurons form networks that are plastic in nature. The spiking dynamics of neurons, the weight (transmission efficiency) of the synaptic contacts, and the structure of the networks can change with time [1–4]. The plastic nature of neuronal networks enables learning and memory [5, 6], stabilization of networks in spontaneous and experience-induced conditions [7, 8], and recovery and rehabilitation after stroke and injuries [9–11]. Alterations in neural plasticity may support pathological conditions such as Parkinson’s disease (PD) [12, 13] and epilepsy [14].

Models of plastic neuronal networks are used to study the functioning of specific brain areas in healthy and disordered conditions and to develop therapeutic stimulation methods [15–20]. Plastic networks of leaky integrate-and-fire (LIF) model neurons have been used to design and validate effective stimulation methods such as coordinated and random reset (CR and RR) stimulation [21–24], which leverage the synaptic weight plasticity to induce therapeutic effects.

The weight of a synaptic contact can change depending on the exact timing of the spikes of the pre- and post-synaptic neurons [25, 26]. This mechanism is termed STDP [26–28]. Networks of LIF neurons with STDP can display bistablily by residing in either a synchronized or a desynchronized state [21–24], which mimic the pathological and physiological states, respectively, in the subthalamic nucleus (STN) and Basal Ganglia of patients with PD [29–31]. Stimulation can be employed to counteract abnormal synchrony and induce long-term desynchronization [24, 32–34]. In the case of Alzheimer’s Disease (AD), the desynchronized spiking and decoupling of neurons is observed during disease progression [35, 36]. The re-synchronization of the fast-spiking interneurons restores the gamma oscillations in the hippocampus, reducing the impairment of cognitive function in AD patients [37].

SP is another noted form of plasticity that reorganizes the network structure via addition and elimination (pruning) of synaptic contacts depending on the activity of the neurons, referred to as synaptic reorganization, besides sprouting and reshaping of synaptic elements [4, 6]. The addition and pruning of synaptic contacts may depend on several factors, such as the synaptic weight, firing rates of the neurons, and the physical distance between neurons [4, 38, 39]. In particular, weaker synaptic contacts are more prone to pruning than the strong ones [38, 40–42]. The form of SP that may add or remove synaptic contacts to maintain a homeostatic set-point of the firing rate of the neurons is termed homeostatic SP [4, 6].

Neurons extend their neurites (axons and dendrites), which may reach each other to form potential synaptic contacts [43, 44]. In principle, axons and dendrites of any two neurons may form potential synaptic contacts at multiple locations, some of which may turn into actual synaptic contacts as the synaptic elements (axonal boutons and dendritic spines) bridge the gap between the axons and the dendrites [43, 45]. On the one hand, it allows for the formation of multiple contacts between a pair of neurons [43, 46, 47]. On the other hand, it makes nearby neurons more likely to connect, producing distance-dependent connectivity [48]. Structural changes in a network occur at a much longer timescale than synaptic weight change (due to STDP) and neuronal spiking dynamics [4, 6].

Activity-dependent changes in the structure of a network have been implemented in several studies that aimed to reproduce experimentally observed network behaviors and statistics of connectivity besides identifying the mechanisms underlying the synaptic reorganization of network [6, 46, 47, 49]. Synchrony of a network of non-identical oscillators can be enhanced by employing specific alterations in the network structure [50, 51] and in networks of FitzHugh Nagumo model neurons, synchrony can be enhanced by a combination of STDP and homeostatic SP (hSP) [52]. In networks of oscillators that may represent certain oscillatory neuronal networks, the synaptic reorganization could significantly affect the network dynamics and its response to stimulation compared to networks with fixed structure [53].

The structural properties of the network have been studied using tools from network science, such as node degree distribution and correlation, clustering coefficient, average path length, and assortativity [54–56]. Networks of oscillators that evolve with SP show degree-frequency correlations, while such correlations may be absent in random graphs [50, 53]. The assortativity is linked to certain network properties, e.g. stability, robustness, and information content [56, 57]; real-world networks, such as neural networks and co-authorship networks are either assortative or dissasortative [56]. In practice, multiple measures are often used together as one metric may not suffice to describe and distinguish the structural properties of different networks. In the present study, we employ degree distribution, degree-frequency and degree-degree correlations, and degree assortativity measures to fully describe the network structure and distinguish between the structure that emerges due to synaptic reorganization in the presence of SP and that of random networks.

The timescales of neuronal spiking and changes in synaptic weights and network structure are rather distinct. Whereas the spiking activity may occur at a sub-second timescale, the synaptic weight and structural changes may take minutes to hours [58] and hours to days [59, 60], respectively. Incorporating all three in a computational study thus poses the challenge of maintaining such distinct timescales while keeping the network model computationally less costly so that a detailed analysis of the network dynamics may be conducted. Here, we combine the LIF neuron model with a standard additive STDP rule and introduce a stochastic SP rule that adds and eliminates synaptic contacts based on the firing rate of a postsynaptic neuron and the weight of the contacts. The network model maintains the distinction between the timescales of neuronal activity and plasticity mechanisms. The stochastic SP method is computationally fast and we compare it with a prevailing method introduced in Ref. [61], referred to as Butz and van Ooyen SP (BvOSP) in this study, which is neuroscientifically informed but computationally costlier [20]. We show that our stochastic SP method produces network dynamics similar to those by BvOSP. We aim to study the effect of the two distinct plasticity mechanisms (synaptic weight and structural) on the dynamical states of the network and to understand the co-evolution of network activity and structure (synaptic reorganization).

As shown computationally, stimulus responses of neural networks with STDP may significantly differ from stimulus responses in networks with fixed coupling strength, i.e., fixed synaptic weights [21, 23, 24, 33, 62, 63]. STDP may cause a multistability [64, 65], and properly designed stimuli may move networks from one attractor to another, qualitatively different attractor, in this way causing long-term stimulus effects that persist after cessation of stimulation [21, 23, 24, 33, 62, 63]. In general, stimulus-induced reshaping of adaptive systems may have various applications in various fields of applications [66]. For instance, in a clinical context, fundamental predictions derived from stimulated networks with STDP were key for the development of novel therapies: Deep brain stimulation (DBS) is an established treatment for Parkinson’s disease [67, 68]. While being the gold standard for the treatment of medically refractory Parkinson’s disease, DBS still has therapeutic limitations and may cause significant side effects [68]. For DBS, electrical stimuli are permanently and periodically delivered to specific target areas in the brain at rates greater than 100 Hz [67–69]. To specifically counteract Parkinson’s-related abnormal neuronal synchrony by desynchronization, Coordinated Reset (CR) stimulation, a patterned multi-channel stimulation technique, was computationally developed [70]. To overcome the need for permanent stimulation, based on computational studies in neural networks with STDP, it was suggested to use CR stimulation to cause long-lasting desynchronization by an unlearning of abnormal synaptic connectivity [33, 62, 63]. Non-trivial qualitative stimulus-response predictions, e.g., the emergence of cumulative and long-term effects [33, 63], were key to the development of the corresponding pre-clinical and clinical experimental and study protocols in the context of Parkinson’s, epilepsy and binge alcohol consumption [71–73]. Furthermore, these stimulus responses enabled the development of non-invasive CR stimulation techniques, e.g., for the vibrotactile treatment of Parkinson’s [74, 75] or the acoustic treatment of chronic subjective tinnitus [76–78].

To account for additional plasticity mechanisms, in this study, we also focus on the effect of SP on the response of neuronal networks to stimulation by comparing the desynchronization of random networks with frozen structures with those that undergo synaptic reorganization to reach a steady synchronized state. To this end, we use our model of plastic neuronal networks to assess the effectiveness of a stimulation protocol named Uncorrelated Multichannel Random Stimulation (UMRS), a technique similar to previously developed stimulation methods belonging to the CR family [21, 33, 79], in driving the network out of a pathological model state thereby inducing long-term desynchronization.

## Model and methods

We use excitatory networks of conductance-based LIF neurons developed in [21, 79] to model synchronized and desynchronized states in the subthalamic nucleus. The unconnected neurons fire periodically with natural firing rates randomly distributed around the mean *f*_0_. The standard deviation of the natural firing rates, *σ*_*f*_, serves as a measure of heterogeneity or diversity and is used here as one of the control parameters. When the neurons are randomly connected and the synaptic weights are governed by an additive STDP rule, the network may settle in either a synchronous or asynchronous state, depending on the initial distribution of synaptic weights. We include SP in the network model, which allows time-dependent changes in the network structure according to the neuronal activity and the synaptic weights. We develop a stochastic model of SP in which synaptic contacts are modeled with a birth-death process (governing addition or pruning of contacts) with corresponding rates that depend on the firing rates of neurons and on the synaptic weights of the contacts. The maximal probabilities of addition and pruning are other control parameters of our model. We use an adiabatic approach for the SP dynamics to overcome the challenge of the diverse time scales of neuronal firing, STDP-induced weight dynamics, and extremely slow SP. In this approach, the network is assumed to reach a meta-steady state in between consecutive structural updates.

### Network model

We place *N* = *m* × *m* neurons on a regular square lattice of size *L* with spacing *h* = *L*/(*m* − 1). The spatial coordinates of a given neuron *i* that lies at lattice index (*i*_*x*_, *i*_*y*_) are

$$\begin{array}{c} x_{i}=\frac{h}{2}(2i_{x}-1)+\xi_{x_{i}},\;y_{i}=\frac{h}{2}(2i_{y}-1)+\xi_{y_{i}},\;i_{x},i_{y}=1,...,m. \end{array}$$ (1)

$\xi_{x_{i}}$ and $\xi_{y_{i}}$ are small random jitters in the x- and y- coordinates of the neurons. The neurons are enumerated as *i* = *i*_*x*_ + (*i*_*y*_ − 1)*m*, where *i* = 1, …, *N*.

The membrane potential of *i*-the neuron, is governed by [21]

$$\begin{array}{c} C\frac{dV_{i}\left(t\right)}{dt}=g_{\text{leak},i}\left(V_{\text{rest}}-V_{i}\left(t\right)\right)+g_{\text{syn},i}\left(t\right)\left(V_{\text{syn}}-V_{i}\left(t\right)\right)+I_{\text{stim},i}\left(t,l_{i,r}\right)+I_{\text{noise},i}\left(t\right), \end{array}$$ (2)

where *C* is the membrane capacitance, *g*_leak,*i*_ is the leak conductance, and *V*_rest_ is the resting membrane potential. The time-varying synaptic conductance, *g*_syn,*i*_(*t*), and the reversal potential, *V*_syn_, determine the time-varying synaptic inputs from the presynaptic partners. *I*_stim,*i*_(*t*, *l*_*i*,*r*_) is the stimulation current received by the neuron *i* at a distance *l*_*i*,*r*_ from the *r*-th stimulation electrode [detailed in the *’Stimulation’* section], which could be set to 0 for all *i* to study the stead-state dynamics of the network. Lastly, *I*_noise,*i*_ represents the noisy inputs from the other sources, e.g., other neuronal populations not included in the model. A neuron fires a spike whenever its membrane potential crosses the dynamic threshold, *V*_th,*i*_, governed by

$$\begin{array}{c} \tau_{\text{th}}\frac{dV_{\text{th},i}\left(t\right)}{dt}=V_{\text{th,rest}}-V_{\text{th},i}. \end{array}$$ (3)

*τ*_th_ is the threshold time constant and *V*_th,rest_ is the resting threshold potential. Once the neuron generates a spike, its membrane potential, *V*_*i*_(*t*), is kept at *V*_spike_ and *V*_th,*i*_(*t*) is kept at *V*_th,spike_ for a duration of *τ*_spike_. After that, the membrane potential is reset to *V*_reset_.

The synaptic conductance, *g*_syn,*i*_, follows

$$\begin{array}{c} \tau_{\text{syn}}\frac{dg_{\text{syn},i}\left(t\right)}{dt}=-g_{\text{syn},i}\left(t\right)+\kappa \frac{\tau_{\text{syn}}}{N}\sum_{j=1}^{N}\sum_{k=1}^{A_{i,j}\left(t\right)}w_{i,j,k}\left(t\right)\sum_{\mu }\delta \left(t-t_{j,\mu }-t_{d}\right). \end{array}$$ (4)

Here, *τ*_syn_ is the synaptic time constant, *κ* is the maximal coupling strength, *t*_*j*,*μ*_ is the timing of the *μ*-th spike of the *j*-th presynaptic neuron, and *t*_*d*_ is the synaptic time delay. In general, multiple contacts can exist from neuron *j* to neuron *i*, the weights of which are given by the elements, *w*_*i*,*j*,*k*_(*t*), of the N×N×M weight matrix, **W**(t), where M is the maximum number of contacts permitted between any pair of pre- and post-synaptic partners, and index *k* refers to the specific contact from neuron *j* and neuron *i*. The sum over *k* in Eq 4 refers to the summation over *A*_*i*,*j*_ contacts, where *A*_*i*,*j*_(*t*) is the element of the *N* × *N* adjacency matrix, **A**(t). The adjacency matrix determines the network structure such that *A*_*i*,*j*_(*t*) assumes a value >0 if synaptic contacts from neuron *j* to *i* exist, and 0 otherwise. Therefore, index *i* refers to a postsynaptic neuron and *j* to its presynaptic partner. No self-loops are allowed, i.e., *A*_*i*,*i*_(*t*) = 0. The time-dependencies of the weight and adjacency matrices correspond to the weight and structural plasticity, respectively.

The noisy input from other sources is modeled as independent Poisson spike train with the constant rate *f*_noise_, and is given by

$$\begin{array}{c} I_{\text{noise},i}\left(t\right)=g_{\text{noise},i}\left(t\right)\left(V_{\text{syn}}-V_{i}\left(t\right)\right), \end{array}$$ (5)

where the synaptic noise conductance, *g*_noise,*i*_(*t*), follows

$$\begin{array}{c} \tau_{\text{syn}}\frac{dg_{\text{noise},i}\left(t\right)}{dt}=-g_{\text{noise},i}\left(t\right)+\kappa_{\text{noise}}\tau_{\text{syn}}\sum_{\mu }\delta \left(t-t_{i,\mu }\right). \end{array}$$ (6)

In Eq 6, the second term represents Poisson noise with the noise intensity *κ*_noise_. The summation is over Poisson spikes, where *t*_*i*,*μ*_ is the timing of the *μ*-th Poisson spike fed to neuron *i*.

### STDP

We use the additive STDP rule as in Ref. [21]. The change in the weight of the synaptic contacts from a presynaptic neuron *j* to its postsynaptic partner *i* is given by

$$\begin{array}{c} \delta w_{i,j}\left(q\right)=\eta \{\begin{array}{cc} \text{exp}(-\frac{\left|q\right|}{\tau_{+}}), & q>0,\\ -\frac{b}{\tau_{R}}\;\text{exp}(-\frac{\left|q\right|}{\tau_{R}\tau_{+}}), & q\leq 0, \end{array} \end{array}$$ (7)

Thus, *w*_*i*,*j*,*k*_(*t*) → *w*_*i*,*j*,*k*_ + *δw*_*i*,*j*_(*q*) ∀*k*. Here, *q* = *t*_*i*_ − *t*_*j*_ − *t*_*d*_ is the time lag between the spikes of the neurons *i* and *j*. *η* ≪ 1 ensures a longer timescale of synaptic weight change compared to neuronal membrane potential dynamics. *τ*_+_ is the long-term potentiation (LTP) time constant and *τ*_*R*_ scales the long-term depression (LTD) time constant relative to *τ*_+_. The total synaptic weight change due to all possible values of time lag, $\int_{-\infty }^{\infty }\delta w_{i,j}\left(q\right)dq=\eta \tau_{+}\left(1-b\right)$. Thus, *b* determines the asymmetry of the STDP rule, such that *b* < 1 makes STDP potentiation dominant, *b* > 1 makes it depression dominant, and *b* = 1 makes it balanced. The parameters of the STDP rule, including the time delay *t*_*d*_ are kept the same as in Refs. [21, 79], given in Table 1. Eq 7 is implemented as a set of differential equations for the weight matrix, **W**(t), and the traces *χ*(*t*), *ψ*(*t*) for pre- and post-synaptic spike trains [80],

$$\begin{array}{ccc} & & {\dot{w}}_{i,j}=\eta [\chi_{j}\left(t\right)\;\delta \left(t-t_{i}\right)-\frac{b}{\tau_{R}}\;\psi_{i}\left(t\right)\;\delta \left(t-t_{j}-t_{d}\right)],\\ & & {\dot{\chi }}_{j}=-\frac{1}{\tau_{+}}\;\chi_{j}+\sum_{\mu }\delta \left(t-t_{j,\mu }-t_{d}\right),\;{\dot{\psi }}_{i}=-\frac{1}{\tau_{R}\tau_{+}}\;\psi_{i}+\sum_{\mu }\delta \left(t-t_{i,\mu }\right). \end{array}$$ (8)

where *t*_*i*,*μ*_ is the timing of the *μ*-th spike of the postsynaptic neuron and *t*_*j*,*μ*_ is that of its presynaptic partner. *t*_*i*_ and *t*_*j*_ represent the latest spike times that trigger potentiation and depression, respectively.

**Table 1:** Parameters of the network model and stimulus.

Integrate-&-fire model parameters	value
Resting membrane potential, *V*_rest_	-38 mV
*V*_reset_	-67 mV
Resting threshold potential, *V*_th,rest_	-40 mV
Reversal potential, *V*_syn_	0 mV
*V*_spike_	20 mV
*V*_th,spike_	0 mV
Synaptic time delay, *t*_*d*_	3 ms
Threshold potential time constant, *τ*_th_	5 ms
Synaptic input time constant, *τ*_syn_	1 ms
Duration of a spike, *τ*_spike_	1 ms
Membrane capacitance, *C*	3 *μ*F/cm^2^
Maximal coupling strength, *κ*	8 mS/cm^2^
Average natural firing rate, *f*_0_	3 Hz
Noisy input rate, *f*_noise_	20 Hz
Noise intensity, *κ*_noise_	0.06 mS/cm^2^
**STDP parameters**	
LTP time constant, *τ*_+_	10 ms
Scaling parameter for LTD, *τ*_*R*_	4 ms
Maximum change in weight, *η*	0.02
Asymmetry, *b*	1.4
**SP parameters**	
Maximal homeostatic SP probability, *P*_*h*_	0.01
Maximal weight-dependent pruning probability, *P*_w_	varied
Probability decay constant (with distance), *l*_0_	0.5 L
Δ*f*	1 Hz
Target firing rate, *f*_T_	4.5 Hz
Minimum value of logistic function, ${\tilde{p}}_{T}$	0.01
*w*_min_	0.001
*τ*_slow_	30 minutes
**Stimulus parameters**	
Maximum stimulus intensity, *I*_0_	402 *μ*A/cm^2^
Duration of positive pulse, $\tau_{s}^{p}$	0.5 ms
Duration of negative pulse, $\tau_{s}^{n}$	1.5 ms
Gap between positive and negative pulses, $\tau_{s}^{g}$	0.2 ms
Fraction of neurons stimulated by one electrode, *γ*	0.2
SD of stimulus Gaussian profile, *σ*_*s*_	0.084

### SP

Computational models of SP may include all aspects, from neurite outgrowth and retraction [81] to the generation and deletion of synaptic elements to the probabilistic formation of synaptic contacts [61]. BvOSP [49, 61] is a model that generates and deletes synaptic elements based on the average activity level of neurons and makes them available for synapse formation without modeling the activity-dependent outgrowth or retraction of neurites in order to form contacts and allows for multiple contacts between a pair of pre- and post-synaptic neurons, i.e., the adjacency matrix elements, *A*_*i*,*j*_ ∈ {0, 1, 2, ‥}. Its algorithm is presented in detail in the S1 Text and Refs. [49, 61, 82]. It has been used to generate networks from completely unconnected neurons spontaneously, reproduce experimentally observed network reorganization after lesion [49, 82], and explain clinically observed therapeutic effects of CR [20], transcranial direct current stimulation [83], and transcranial magnetic stimulation [84]. In a recent study, BvOSP was used to show that hSP is responsible for biphasic changes in the connectivity of pyramidal neurons in mouse anterior cingulate cortex 24 and 48 hours after optogenetic stimulation [85].

The inclusion of BvOSP in computational studies aimed at investigating the evolution of networks with both weight and structural plasticity over long periods of time may require considerably long computation time, which could limit the extent to which the impact of SP and variations in its control parameters on the network dynamics and properties can be studied. Establishing a simpler model of SP that captures the essential mechanisms that govern changes in brain networks may allow for a reliable and more detailed numerical analysis. We propose a simple model, stochastic SP (described below), which directly builds or eliminates synaptic contacts between neurons without separately modeling the neurite outgrowth/retraction or generation/deletion of synaptic elements. We further simplify the model by allowing only a single synaptic contact from a given presynaptic neuron to its postsynaptic partner that accounts for its overall effect on the postsynaptic one, i.e., *A*_*i*,*j*_ ∈ {0, 1}, and M=1, although it can be easily extended to include multiple synaptic contacts between a pair of pre- and post-synaptic neurons. We validate the stochastic SP method by comparing our results with those obtained using BvOSP, where we allow for multiple contacts with M=10 so that *A*_*i*,*j*_ ∈ {0, 10}.

We base our stochastic SP model on the experimental evidence stated above. The probability of the addition of a synaptic contact between two neurons, *P*_add_, depends on the Euclidean distance between them and the firing rate of the postsynaptic neuron. Primarily, the postsynaptic neurons with firing rates below the homeostatic set-point (target) firing rate develop synaptic contacts with nearby neurons. The synaptic contacts are pruned depending on both synaptic weight and the firing rate of postsynaptic neurons, which together determine the pruning probability, *P*_prn_. In particular, the synaptic contacts that are weaker or deliver inputs to neurons with a firing rate above the target firing rate are more likely to be pruned, consistent with experimental observations [41, 42]. The addition of a synaptic contact from a neuron *j* to *i* changes the adjacency matrix element *A*_*i*,*j*_ from 0 to 1 while pruning changes *A*_*i*,*j*_ from 1 to 0. The newly added contacts are given a small random weight, following experimental evidence [41].

The dependence of the pruning and addition probabilities on the postsynaptic neuron’s firing rate models a homeostatic process whereby the neuron’s activity is maintained at a target firing rate, *f*_T_. The probability of addition of incoming contacts increases with a decrease in the neuron’s firing rate below *f*_T_, while its pruning probability increases with an increase in the neuron’s firing rate above *f*_T_. The firing rate of the *i*-th neuron is calculated by low-pass filtering its spike train as,

$$\begin{array}{c} \tau_{\text{slow}}{\dot{f}}_{i}\left(t\right)=-f_{i}\left(t\right)+\sum_{\mu }\delta \left(t-t_{i,\mu }\right), \end{array}$$ (9)

where *t*_*i*,*μ*_ is the timing of the *μ*-th spike of *i*-th neuron and *τ*_slow_ is the time constant.

The process of addition is purely homeostatic, i.e., weight-independent. The probability of addition is given by

$$\begin{array}{c} P_{\text{add}}\left(f_{i},l_{i,j}\right)=P_{h}\;G\left(f_{i},f_{-},-\nu \right)\;\text{exp}\;(-\frac{l_{i,j}}{l_{0}}), \end{array}$$ (10)

where *P*_*h*_ is the maximal probability of the homeostatic SP (both addition and pruning), *l*_0_ is the decay constant for distance dependence, and $l_{i,j}=\sqrt{{\left(x_{i}-x_{j}\right)}^{2}+{\left(y_{i}-y_{j}\right)}^{2}}$ is the Euclidean distance between neurons *i* and *j*. Since the neurons are arranged on *L* × *L* square lattice, the network structure becomes distance-independent for $l_{0}\gg \sqrt{2}L$. The firing rate dependence function, *G*(.), is a logistic function defined as

$$\begin{array}{c} G\left(\Omega ,\Omega_{0},\nu \right)=\frac{1}{1+\text{exp}(-\frac{\Omega -\Omega_{0}}{\nu })}, \end{array}$$ (11)

where the parameters Ω_0_ and *ν* determine the midpoint and the slope, respectively.

In our model, a synaptic contact can be pruned independently by either the homeostatic or weight-dependent processes. The homeostatic component of the pruning is given by

$$\begin{array}{c} P_{\text{prn, h}}\;\left(f_{i}\right)=P_{h}\;G\left(f_{i},f_{+},\nu \right). \end{array}$$ (12)

We assume non-zero probabilities of addition and pruning when the firing rate of a post-synaptic neuron matches its target rate, *f*_T_, i.e., $G\left(f_{\text{T}},\;f_{\pm },\;\nu \right)={\tilde{p}}_{\text{T}},\;{\tilde{p}}_{\text{T}}\ll 1$. The steepness of the logistic function is characterized by the parameter Δ*f*, such that $G\left(f_{\text{T}}-\Delta f,\;f_{-},\;\nu \right)=1-{\tilde{p}}_{\text{T}}$, and $G\left(f_{\text{T}}+\Delta f,\;f_{+},\;-\nu \right)={\tilde{p}}_{\text{T}}$, for pruning and addition, respectively. This gives

$$\begin{array}{ccc} & & f_{\pm }=f_{\text{T}}\pm \frac{\Delta f}{2},\;\nu =\frac{\Delta f}{2\;\text{ln}[\left(1-{\tilde{p}}_{\text{T}}\right)/{\tilde{p}}_{\text{T}})]}, \end{array}$$

The weight-dependent component of pruning is of the form

$$\begin{array}{c} P_{\text{prn},\text{w}}\left(w_{i,j,k}\right)=P_{\text{w}}\;\text{exp}\;(-\frac{w_{i,j,k}}{w_{\text{min}}}), \end{array}$$ (13)

where *w*_min_ ≪ 1 and *P*_w_ is the maximal probability of weight-dependent pruning. The total probability of pruning is,

$$\begin{array}{c} P_{\text{prn}}\left(f_{i},w_{i,j,k}\right)=P_{\text{w}}\;\text{exp}\;(-\frac{w_{i,j,k}}{w_{\text{min}}})+P_{h}\;G\left(f_{i},f_{+},\nu \right). \end{array}$$ (14)

**Fig 1:**
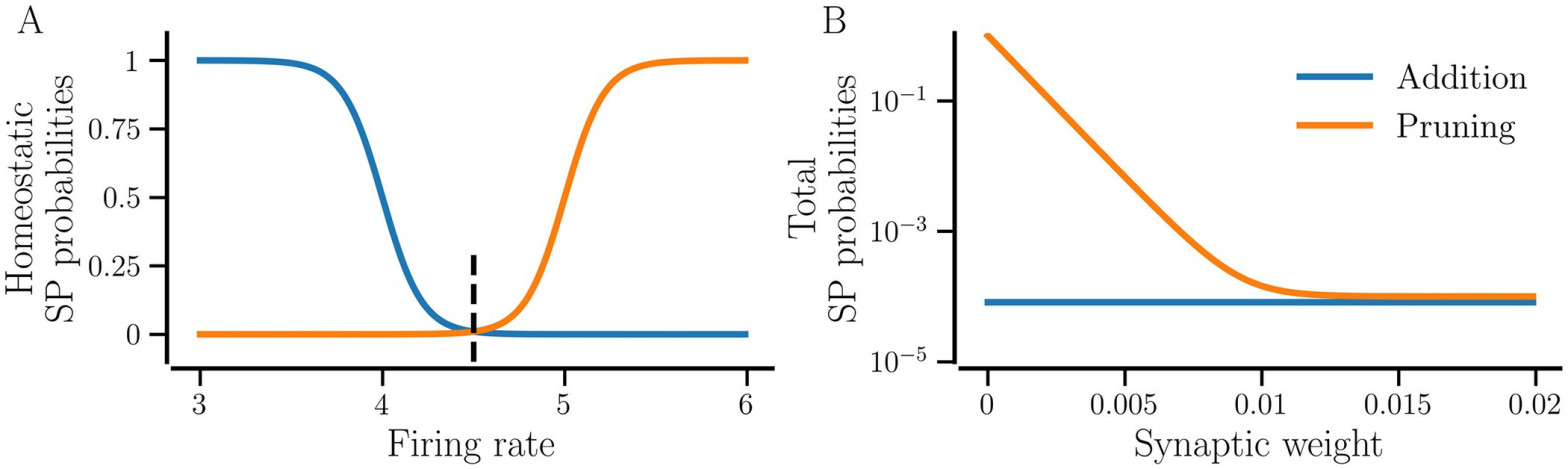
SP probabilities for the typical parameter values. A: Probabilities of homeostatic SP versus the firing rate of a post-synaptic neuron. The vertical dashed line marks the target firing rate, *f*_T_. B: Total SP probabilities versus synaptic weight according to Eqs 10 and 14 when the firing rate of the neuron equals the target rate. The parameters are: Δ*f* = 1 Hz, ${\tilde{p}}_{\text{T}}=0.01$, *f*_T_ = 4.5 Hz, *w*_min_ = 0.001, *l* = 0.1, *l*_0_ = 0.5; *P*_*h*_ = 1 for A and *P*_*h*_ = 0.01, *P*_w_ = 1 for B.

Fig 1 exemplifies the SP probabilities. Since only one contact can be built from neuron *j* to *i*, in our stochastic SP model, the subscript *k* in Eqs 13 and 14 can be dropped.

### Measures

The degree of synchrony of a network is measured using the **Kuramoto order parameter** [21, 86],

$$\begin{array}{c} R\left(t\right)=\frac{1}{T}\int_{t-T}^{t}dt^{\prime }|\frac{1}{N}\sum_{i=1}^{N}e^{\iota \phi_{i}\left(t^{\prime }\right)}|, \end{array}$$ (15)

where *ϕ*_*i*_(*t*′) is the phase of *i*-th neuron that is calculated using the timings of two consecutive spikes, *t*_*i*,*μ*_ and *t*_*i*,*μ* + 1_,

$$\phi_{i}\left(t^{\prime }\right)=2\pi (\frac{t^{\prime }-t_{i,\mu }}{t_{i,\mu +1}-t_{i,\mu }}+\mu ).$$

R(t) ranges from 0 for complete absence of synchrony to 1 for perfect synchrony.

We calculate the **network-averaged firing rate**, 〈*f*〉(*t*) = (1/*N*)∑_*i*_ *f*_*i*_(*t*) and **the coefficient of variation**, CV(*t*), of firing rate defined as the ratio of the standard deviation of firing rate, $\sigma_{f}^{2}\left(t\right)=\left(1/N\right)\sum_{i}{\left(f_{i}\left(t\right)-\langle f\rangle \left(t\right)\right)}^{2}$, to the mean, 〈*f*〉(*t*): CV(*t*) = *σ*_*f*_(*t*)/〈*f*〉(*t*). The combination of order parameter and CV of the firing rate can be used as indicators of synchrony and frequency locking for non-identical oscillators. CV = 0, combined with the order parameter close to 1 indicates a perfectly frequency-locked state. Non-zero CV values indicate non-identical frequencies, e.g., due to incomplete synchronization and/or cluster states with different frequencies.

The synaptic weights are studied using the **average incoming synaptic weight**, *W*_*i*_(*t*), of individual neurons and the **network-averaged synaptic weight**, 〈*W*〉(*t*)

$$\begin{array}{c} \left\langle W\right\rangle \left(t\right)=\frac{1}{N}\sum_{i=1}^{N}W_{i}\left(t\right),\;W_{i}\left(t\right)=\frac{\sum_{j=1}^{N}\sum_{k=1}^{A_{i,j}\left(t\right)}w_{i,j,k}\left(t\right)}{\sum_{j=1}^{N}A_{i,j}\left(t\right)}. \end{array}$$ (16)

〈*W*〉(*t*) ∈ [0, 1], where 0 indicates an uncoupled network and 1 a strongly coupled one. 〈*W*〉(*t*) = 0 (1) may indicate desynchrony (synchrony). *W*_*i*_ → 1 (0) if neuron *i* is strongly (weakly) driven by its presynaptic partners via its incoming contacts. We also study the distribution of the synaptic weight of individual contacts in the steady synchronized states of the network.

To analyze the structure of the network, we use incoming and outgoing node degree densities (**in-NDD and out-NDD**) of individual neurons, given by

$$\begin{array}{c} \beta_{i}^{\text{in}}\left(t\right)=\frac{1}{N}\sum_{j=1}^{N}A_{i,j}\left(t\right)\;\text{and}\;\beta_{j}^{\text{out}}\left(t\right)=\frac{1}{N}\sum_{i=1}^{N}A_{i,j}\left(t\right),\;\text{respectively,} \end{array}$$ (17)

and the **network-averaged NDD**, $\beta \left(t\right)=\frac{1}{N}\sum_{i=1}^{N}\beta_{i}^{\text{in}}\left(t\right)=\frac{1}{N}\sum_{j=1}^{N}\beta_{j}^{\text{out}}\left(t\right)$. *β*(*t*) can range from 0 for a fully unconnected network to 1, marking an all-to-all connected network. For ease of use, we drop the superscript for the in-NDD hereafter, i.e., $\beta_{i}^{\text{in}}\left(t\right)\to \beta_{i}\left(t\right)$.

We employ the **Pearson correlation coefficient** to characterize the **assortativity** of the networks [56, 87, 88]. In (dis)assortative networks, the nodes (here, neurons) tend to connect to other nodes with (dis)similar properties on average. We determine the (dis)assortativity of a network for in- and out-degrees of the neurons as follows [89]. Let *ϵ*, *υ* ∈ {in, out} be the degree-type and $U_{e}^{\epsilon }$ and $V_{e}^{\upsilon }$ be the corresponding degrees of the pre- and post-synaptic neurons, respectively, connected by the *e*-th edge (contact). The Pearson correlation coefficient is given by

$$\begin{array}{c} \rho \left(\epsilon ,\upsilon \right)=\frac{1}{E\sigma^{\epsilon }\sigma^{\upsilon }}\sum_{e=1}^{E}\left[\left(U_{e}^{\epsilon }-{\bar{U}}^{\epsilon }\right)\left(V_{e}^{\upsilon }-{\bar{V}}^{\upsilon }\right)\right], \end{array}$$ (18)

where *E* = *βN*(*N* − 1) is the total number of contacts. The averages, ${\bar{U}}^{\epsilon }=E^{-1}\sum_{e=1}^{E}U_{e}^{\epsilon }$ and ${\bar{V}}^{\upsilon }=E^{-1}\sum_{e=1}^{E}V_{e}^{\upsilon }$. The standard deviations, $\sigma^{\epsilon }={\left[E^{-1}\sum_{e=1}^{E}{\left(U_{e}^{\epsilon }-{\bar{U}}^{\epsilon }\right)}^{2}\right]}^{\frac{1}{2}}$ and $\sigma^{\upsilon }={\left[E^{-1}\sum_{e=1}^{E}{\left(V_{e}^{\upsilon }-{\bar{V}}^{\upsilon }\right)}^{2}\right]}^{\frac{1}{2}}$. We calculate the Pearson correlation coefficient between the in-NDDs of the pre- and post- synaptic neurons (in-in), the out-NDDs (out-out), and between the in-NDD of presynaptic neurons and the out-NDD of postsynaptic neurons (in-out) and vice versa (out-in) [89–91].

Negligible time variations in the network-averaged measures characterize the steady states. We use the network-averaged firing rate, 〈*f*〉, and synaptic weight, 〈*W*〉, to determine the approach to a steady state as follows. We integrate the model equations in time intervals of *T* = 60 s until both 〈*W*〉 and 〈*f*〉 converge with a given relative accuracy, *Υ*,

$$\begin{array}{c} 2\frac{\left|\langle W[nT]\rangle -\langle W[(n+1)T]\rangle \right|}{\left\langle W[nT]\rangle +\langle W[(n+1)T]\right\rangle }<ϒ\;\text{and}\;2\frac{\left|\langle f[nT]\rangle -\langle f[(n+1)T]\rangle \right|}{\left\langle f[nT]\rangle +\langle f[(n+1)T]\right\rangle }<ϒ, \end{array}$$ (19)

where *n* = 1, 2, …, *n*_min_, ‥, *n*_max_ is the number of the convergence intervals (iterations). *n*_min_ is the minimum number of intervals and *n*_*max*_ is the maximum number of iterations required to achieve the relative accuracy of *Υ* = 10^−3^.

### Stimulation

Stimulation is used to induce therapeutic effects in pathological conditions, such as epilepsy [92–94] and Parkinson’s disease [72, 75, 95, 96], where a synchronized state is associated with pathology while incoherent spiking of neurons is observed in a healthy state.

**Fig 2:**
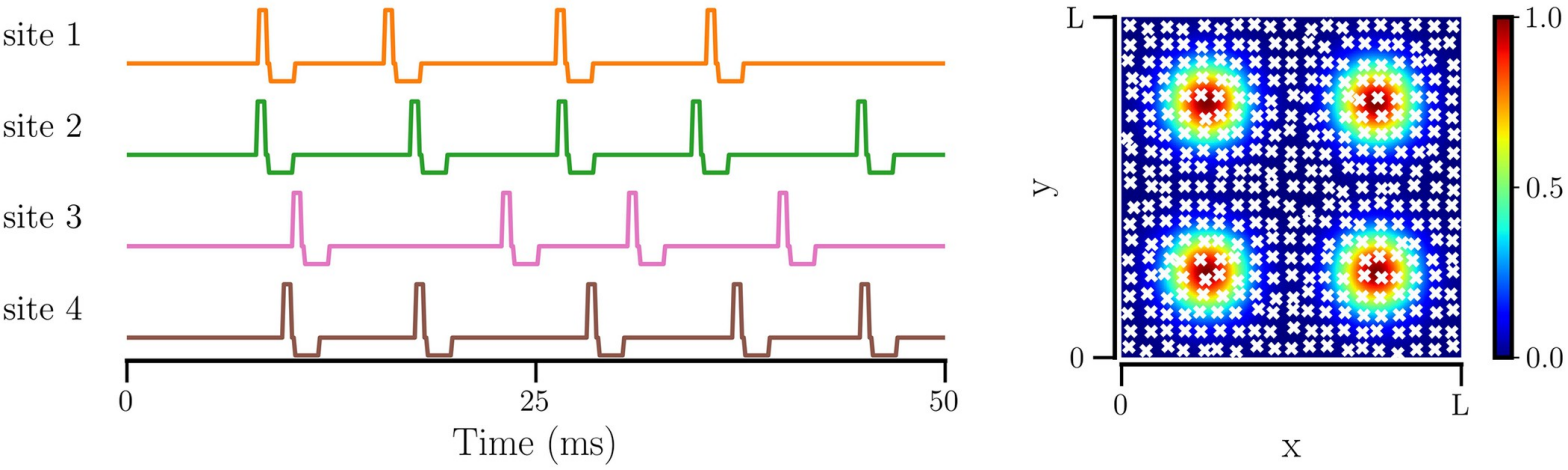
UMRS stimulus administered at *N*_*s*_ = 4 sites. The sites of stimulation are chosen to be the centers of the 4 quadrants in the square network plane. The left panel shows the example stimulus pattern, *X*(*t*), with *F*_*s*_ = 100 Hz for a 50 ms duration. Each site of stimulation receives an independent stimulus and the time interval between stimulus events (each event comprising a positive pulse followed by a negative pulse after a short gap) is exponentially distributed. The right panel shows the spatial variation of stimulus with distance from the electrodes, controlled by *D*(*l*_*i*,*r*_), for all 4 electrodes placed at the centers of four quadrants. Neurons are marked with crosses at their positions.

We use a multichannel stimulation protocol, UMRS (illustrated in Fig 2), where *N*_*s*_ electrodes at fixed locations deliver stimulation independently. Each stimulation site, *r*, receives uncorrelated stimulus at random times with exponentially distributed inter-pulse intervals, similar to the temporal randomness of the RR stimulation [21, 22]. The stimulus from the *r*-th electrode received by neuron *i* at a distance *l*_*i*,*r*_ from the electrode is given by *a*_*s*_*I*_0_*X*_*r*_(*t*)*D*(*l*_*i*,*r*_), where *X*_*r*_(*t*) is the charge-balanced stimulation current of the *r*-th electrode. The dimensionless parameter *a*_*s*_ ∈ [0, 1] scales the magnitude, *I*_0_, of the stimulus, and *D*(*l*_*i*,*r*_) determines the spatial drop in stimulus with distance from the electrode. The total amount of stimulus received by *i*-th neuron is

$$\begin{array}{c} I_{\text{stim},i}\left(t,l_{i,r}\right)=a_{s}I_{0}\sum_{r=1}^{N_{s}}X_{r}\left(t\right)D\left(l_{i,r}\right). \end{array}$$ (20)

The time-dependent charge-balanced stimulus current, *X*_*r*_(*t*), is a random sequence of stimulus events consisting of positive and negative pulses. Each rectangular positive pulse of duration $\tau_{s}^{p}$ and amplitude 1, followed by a short gap, $\tau_{s}^{g}$, and then by a rectangular negative pulse of duration $\tau_{s}^{n}$ and amplitude $-\tau_{s}^{p}/\tau_{s}^{n}$. The value of the magnitude *I*_0_ is chosen as $I_{0}=\left(V_{\text{th,spike}}-V_{\text{reset}}\right)\langle C_{i}\rangle /\tau_{s}^{p}$. Intervals between excitatory pulses are exponentially distributed with the mean inter-pulse interval *τ*_UMRS_. We imposed a minimum interval between subsequent stimulus events at *τ*_Λ_ = 7.692 ms, corresponding to a maximum stimulation frequency of 130 Hz [21, 22]. Thus, the mean stimulation frequency, *F*_*s*_ = (*τ*_UMRS_ + *τ*_Λ_)^−1^.

For scaling the stimulus with distance, we use a Gaussian function, $D\left(l_{i,r}\right)=e^{-l_{i,r}^{2}/2\sigma_{s}^{2}}$, for simplicity. The parameter *σ*_*s*_ determines the area of stimulus spread and can be associated with the number of neurons that effectively receive the stimulus. For a fraction, *γ*, of neurons or the area of the network that we intend to stimulate with each electrode, the value of *σ*_*s*_ can be determined as follows: Assume that below 1% of the maximal intensity, the stimulus is not effective, i.e., it does not affect the spiking of neurons. The area covered by the stimulus from each electrode is *π*(3*σ*_*s*_)^2^ ≈ *γL*^2^, where *L* is the network size, since *D*(*l*_*i*,*r*_) drops to ≈ 0.01 at *l*_*i*,*r*_ = 3*σ*_*s*_. Therefore,

$$\begin{array}{c} \sigma_{s}=\frac{1}{3}\sqrt{\frac{\gamma }{\pi }}L=\frac{1}{3}\sqrt{\frac{\gamma }{\pi }}\left(\sqrt{N}-1\right)h, \end{array}$$ (21)

since $L=\left(m-1\right)h=\left(\sqrt{N}-1\right)h$.

### Parameters and implementation

The parameters of LIF neurons were the same as in [21]. Table 1 contains the values of the model parameters, unless stated otherwise. To generate the Gaussian distributed firing rates of unconnected neurons with the network-averaged firing rate *f*_0_ and SD *σ*_*f*_, the leak conductance of neurons, *g*_leak,*i*_, was drawn from a Gaussian distribution with mean ${\tilde{g}}_{\text{leak}}$ and standard deviation $\sigma_{g_{\text{leak}}}$. The parameter *σ*_*f*_ quantifies the diversity of neurons and was varied in this study. ${\tilde{g}}_{\text{leak}}$ and $\sigma_{g_{\text{leak}}}$ were obtained numerically for a given noise intensity, *κ*_noise_, as follows. First, we calculated and tabulated the long-time average firing rate of a single neuron as a function of *g*_leak_, *f*(*g*_leak_), for values of *g*_leak_ in the range 0.005 ≤ *g*_leak_ ≤ 0.05 mS/cm^2^. Second, we best linear fitted *f*(*g*_leak_), as *f*(*g*_leak_) = *α*_1_*g*_leak_ + *α*_2_. The regression coefficients, *α*_1_ and *α*_2_, were then used to calculate

$$g_{\text{leak}}=\frac{f-\alpha_{2}}{\alpha_{1}}+\alpha_{1}.$$

Now, if *f* is a random Gaussian variable with the mean *f*_0_ and SD *σ*_*f*_, then the equation above can be considered as a linear transformation of *f*. Then, *g*_leak_ is also Gaussian random variable with the mean ${\tilde{g}}_{\text{leak}}$ and SD $\sigma_{g_{\text{leak}}}$ given by

$${\tilde{g}}_{\text{leak}}=\frac{f_{0}-\alpha_{2}}{\alpha_{1}},\;\sigma_{g_{\text{leak}}}=\frac{\sigma_{f}}{\alpha_{1}}.$$

For noise intensity, *κ*_noise_ = 0.06 mS/cm^2^, used in this study, *α*_1_ = 125.67 and *α*_2_ = 0.92.

In this study, we separately consider networks with

1. only synaptic weight plasticity, labeled as *STDP-only*;
2. homeostatic SP (hSP) alone, i.e., without weight-dependent pruning (*P*_w_ = 0) and without STDP, labeled as *hSP-only*;
3. a combination of STDP and hSP, i.e., without weight-dependent pruning (*P*_w_ = 0), labeled as *STDP+hSP*;
4. a combination of STDP and SP that includes hSP and weight-dependent pruning (*P*_w_ ≠ 0), labeled as *STDP+SP*.

For networks with *STDP-only*, the neurons are connected on a random graph with a probability equal to the desired average NDD, i.e., *p* = *β*_0_ = *β*(0), on a square lattice of size *L* = 1 mm. The probability for two neurons to connect decreases exponentially with distance as exp(−*l*/*l*_0_). The initial membrane potentials of the neurons are drawn from a uniform distribution between *V*_reset_ and *V*_rest_. The threshold potential, *V*_th,*i*_ is set to *V*_th,rest_ mV, and *g*_syn,*i*_ and *g*_noise,*i*_ are set to 0 for all neurons. The convergence to steady state is determined using Eq 19, where *n*_min_ is set to 60, and *n*_max_ can be as large as required for the convergence.

SP operates on a timescale much longer than those of STDP and neuronal spiking dynamics. The computation time required to implement structural updates at every time step or in short intervals of a few milliseconds can be significantly long and limit the detailed study of networks [20, 61]. It is crucial to make computation time shorter for thorough variations of parameters to investigate the network dynamics. Therefore, we separate the structural updates from the spiking dynamics and the synaptic weight change by considering structural updates as discrete events, similar to a previously used strategy [53]. Specifically, we assume that between consecutive structural updates (SP iterations) no structural change occurs for a time interval Δ*T*_sp_ and the network evolves either with *STDP-only* or no plasticity. Further, we require the time interval Δ*T*_sp_ to be long enough to let the network settle in a steady state according to the conditions of Eq 19 with *n*_min_ = 2 and *n*_max_ = 31, so that $\Delta T_{\text{sp}}=\tilde{n}T$ where $\tilde{n}\in [n_{\text{min}},\;n_{\text{max}}]$. Thus, the SP time arrow is measured in units of Δ*T*_sp_, *T*_sp,*n*′_ = *n*′Δ*T*_sp_, where *n*′ is the number of SP iterations.

In the following, we consider 20 × 20 networks of *N* = 400 neurons. Larger networks with *N* = 1024 neurons (32 × 32) yielded qualitatively similar results; an example of time evolution of network measures for *N* = 1024 is presented in Fig I in S1 Text. All simulations for spontaneous network dynamics were repeated and averaged for 10 realizations of random networks.

## Results

### Networks with *STDP-only*

**Fig 3:**
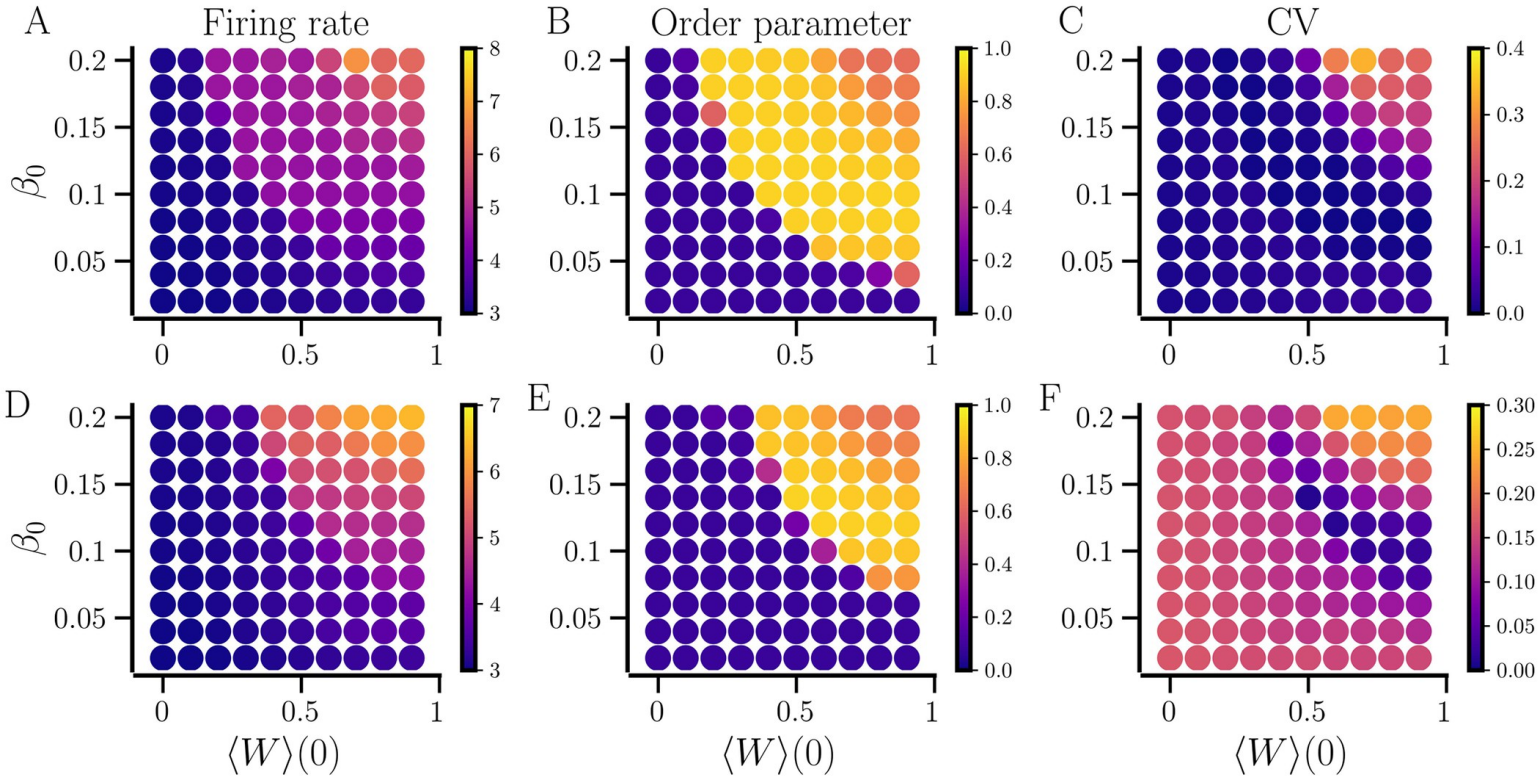
Steady states of the networks of identical (*σ*_*f*_ = 0 Hz, top) and non-identical neurons (*σ*_*f*_ = 0.5 Hz, bottom) with *STDP-only*. The colors indicate the network-averaged values of the firing rate in A and D, the order parameter in B and E, and the CV of the firing rate of neurons in C and F for various values of the initial average synaptic weight, 〈*W*〉(0) and NDD, *β*_0_. The results are averaged over ten realizations of the random networks for each combination of 〈*W*〉(0) and *β*_0_. Other parameters are given in Table 1.

We study the steady-state dynamics that emerge spontaneously in a random network of LIF neurons with *STDP-only* for various initial conditions determined by the initial values of the network-averaged synaptic weight, 〈*W*〉(0), and NDD, *β*_0_. The steady-state measures for the given initial conditions averaged over ten realizations of the random network of identical neurons (*σ*_*f*_ = 0) are shown in Fig 3 (top panels).

**Fig 4:**
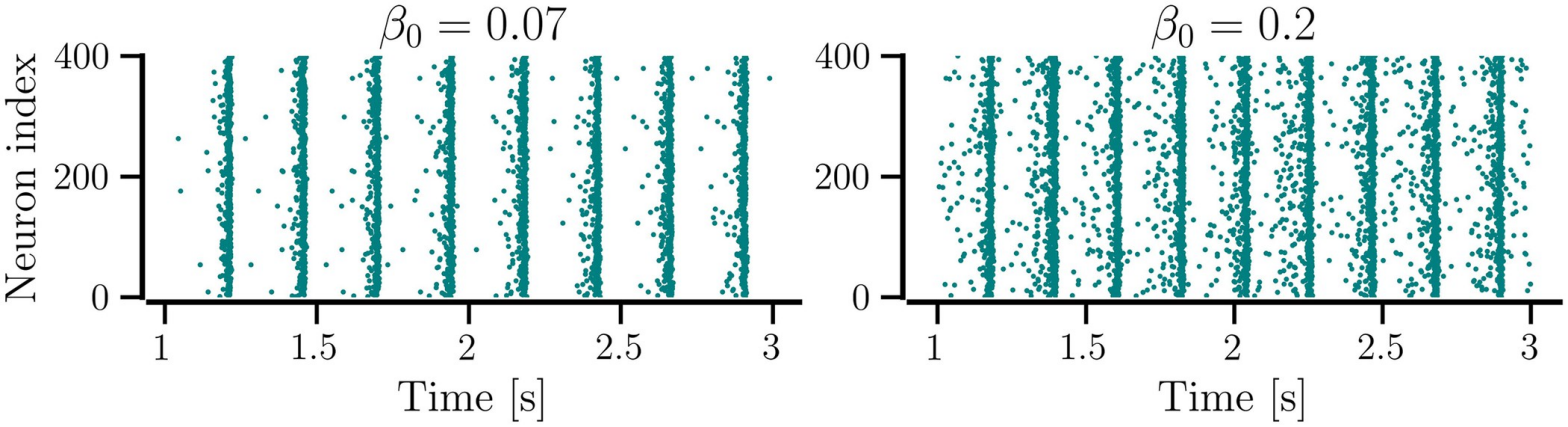
Spike-raster plots of the network of identical neurons with *STDP-only*. The left panel shows the raster plot for average NDD, *β* = 0.07, and the right panel shows the same for *β* = 0.2. For both cases, the network started with an initial average weight of 0.8. Other parameters are given in Table 1.

When the network is sparse, *β*_0_ ≤ 0.05, it settles in a desynchronized state regardless of 〈*W*〉(0). For *β*_0_ ≈ 0.05 and above, the network portrays multistability by settling in either desynchronized, partially synchronized, or fully synchronized states as indicated by the order parameter in Fig 3B. Interestingly, while intermediate values of 〈*W*〉(0) and *β*_0_ produce strongly synchronized states with a minimal CV of firing rates and a high order parameter, larger 〈*W*〉(0) and *β*_0_ cause the network to be only partially synchronized as implied by the higher CV of firing rates in Fig 3C and a lower order parameter in Fig 3B. The raster plot for a synchronized state observed for *β*_0_ = 0.07 in Fig 4 shows minor spike-time jitters while that for a partially synchronized state observed at *β*_0_ = 0.2 it shows significant spike-time jitters, illustrating reduced synchrony with increased *β*_0_ and suggesting the optimal values of NDD for maximum synchrony.

**Fig 5:**
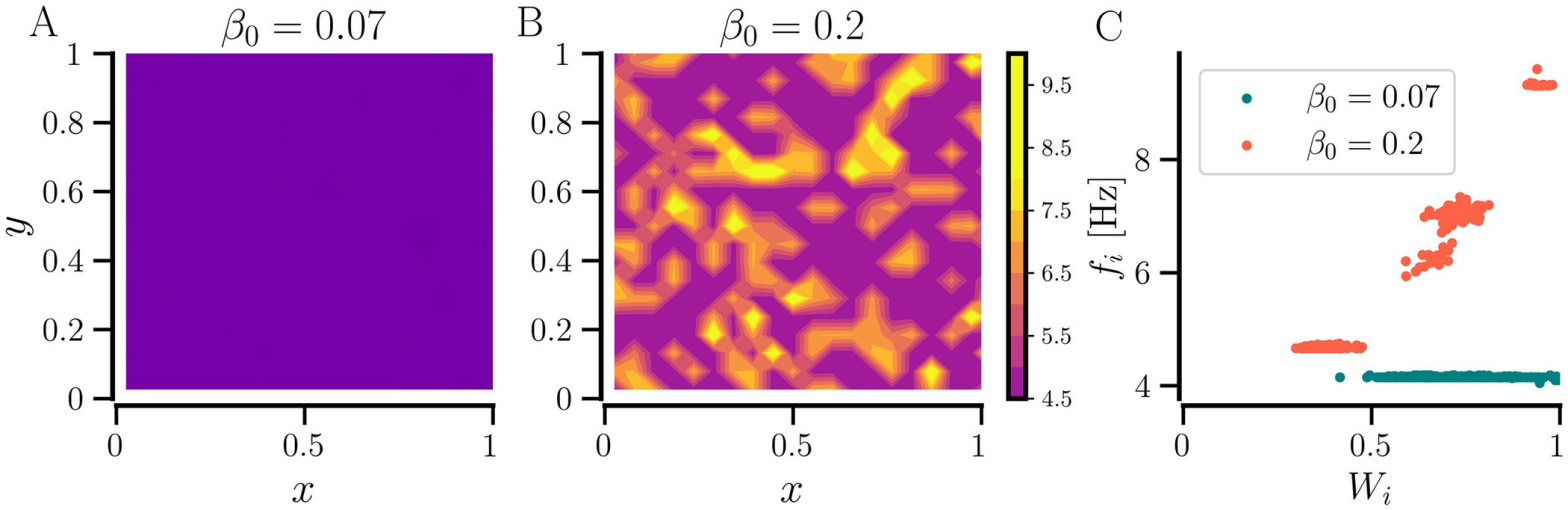
Cluster formation in the network of identical neurons with *STDP-only*. A and B: Time-averaged firing rates versus spatial location of neurons for the two indicated values of average NDD, *β*_0_. C: Time-averaged firing rates versus average synaptic weight. For both cases, the network started with an initial average weight of 0.8. Other parameters are given in Table 1.

The fully synchronized states have a higher network-averaged firing rate than the desynchronized states and even higher firing rates emerge for partially synchronized states, accompanied by higher CV of firing rates. This is caused by the clustering of some neurons at higher firing rates than the network average. Fig 5C shows that the neurons in the denser network (*β*_0_ = 0.2) form 4–5 clusters of the time-averaged firing rate. The firing rates of the clusters depend on their average incoming synaptic weight; naturally, the neurons with larger average incoming weights have higher firing rates. The map of time-averaged firing rates of individual neurons reveals the formation of high-frequency islands for *β*_0_ = 0.2 [Fig 5B] that represent a chimera-like state. The fully synchronized state for *β*_0_ = 0.07 is characterized by a single firing rate ≈ 4.2 Hz, as shown in Fig 5A and 5C.

**Fig 6:**
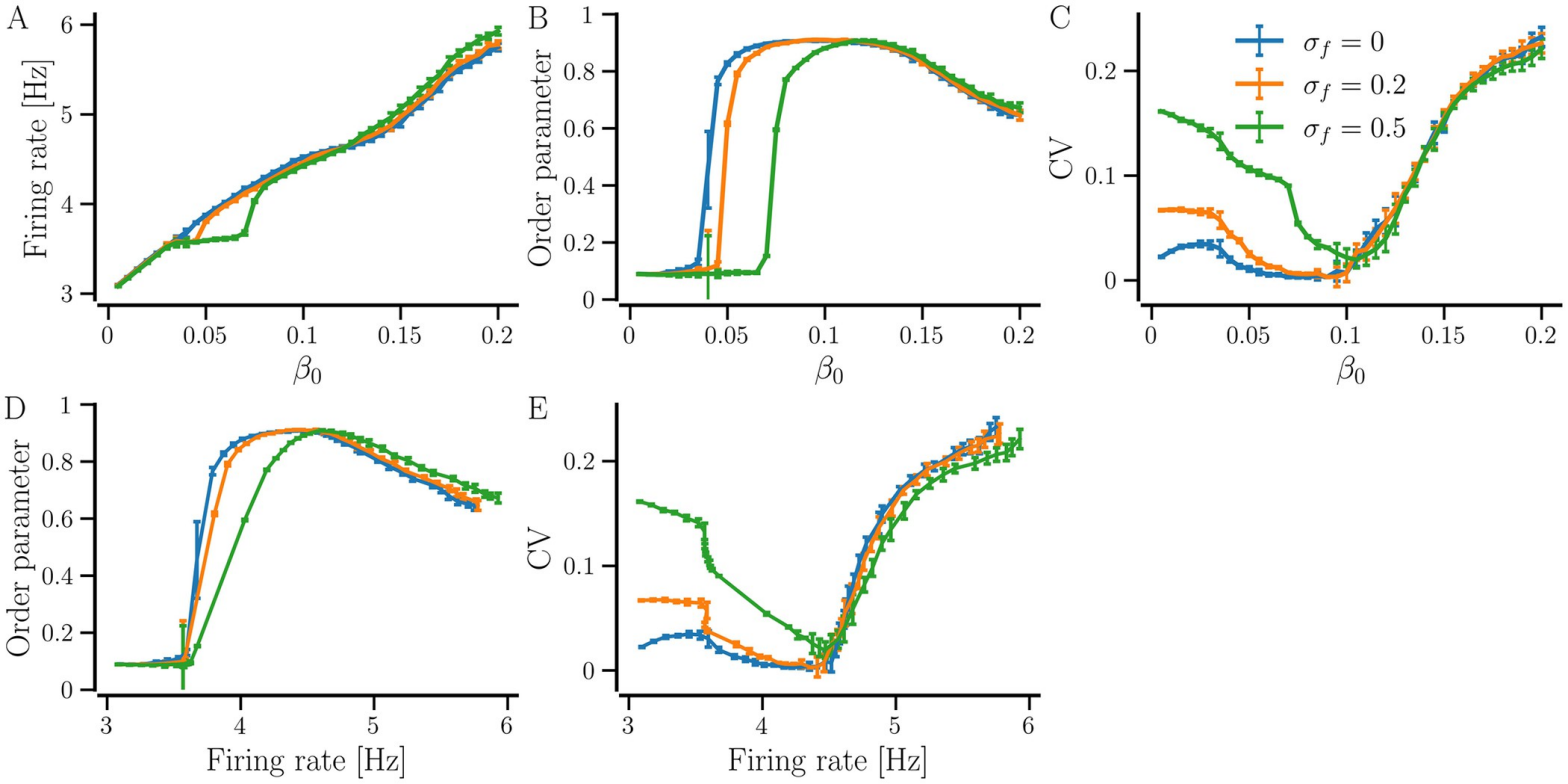
The synchronized state can be optimized by tuning the average NDD for the network with *STDP-only*. A–C: Network-averaged firing rate, order parameter, and firing rate CV versus average NDD, *β*_0_, for the indicated values of diversity parameter, *σ*_*f*_. D and E: Order parameter and firing rate CV versus network-averaged firing rate, obtained as parametric plots of panels A–C. For each value of *β* the network was initialized with the average weight 〈*W*〉_0_ = 0.8 and the results were averaged over 10 realizations of random networks; the error bars show the corresponding standard deviation. Other parameters are given in Table 1.

Next, we study the network of non-identical neurons with *STDP-only*. Neurons are made non-identical in an unconnected state by introducing diversity in the natural firing rates of the neurons, controlled by the diversity parameter, *σ*_*f*_. Similar to the network of identical neurons above, the network of non-identical neurons exhibits multistability as illustrated in Fig 3(bottom panels) where the maps reveal that the ranges of 〈*W*〉(0) and *β*_0_ for which the network settles in a desynchronized state extend to higher values than those for the network of identical neurons due to the diversity in natural firing rates. Nevertheless, the network in synchronized states has a higher network-averaged firing rate than the desynchronized states similar to the network of identical neurons, and the maximum synchrony is observed for intermediate values of 〈*W*〉(0) and *β*_0_ where the firing rate CV is minimum. An increase in 〈*W*〉(0) and *β*_0_ produces partially synchronized states with higher network-averaged firing rate and CV of the firing rate. This is further illustrated in Fig 6A, 6B and 6C. The network-averaged firing rate increases with an increase in the NDD, while the order parameter and the CV of firing rates show a non-monotonous dependence on NDD. Below a critical value of the average NDD, the order parameter resides at a low value, indicating a desynchronized state. Past the critical average NDD, the value of the order parameter increases and reaches a maximum value. A further increase in NDD causes the order parameter to drop to lower values, indicating partial synchrony. The CV of firing rates remains high for small and large values of average NDD and drops to a minimum at intermediate values when the network is strongly synchronized. At small average NDDs, the neurons fail to influence each other and thus, the network does not get synchronized. At larger average NDDs, groups of neurons with larger coupling fire at higher rates than the others, forming clusters and resulting in a high CV of firing rates. The corresponding parametric plots in Fig 6D and 6E show that the maximally synchronized state is obtained at a specific network-averaged firing rate where the maximum order parameter and the minimum firing rate CV are observed coincidentally, indicating maximum phase- and frequency-locking. For example, for *σ*_*f*_ = 0.5 Hz, the network is maximally synchronized at ≈ 4.5 Hz firing rate. A higher firing rate that occurs at a larger average NDD, results in a less coherent state with a higher value of firing rate CV. For a lower *σ*_*f*_, the network gets synchronized with a lower average NDD and firing rate and tends to have a lower CV of firing rates than a network with a higher *σ*_*f*_. The range of average NDD that is large enough to enable synchronization but not enough to promote clustering of neurons is smaller for networks of non-identical neurons compared to those of identical neurons since the identical neurons can get synchronized at significantly lower values of average NDD with a low average firing rate, while clustering occurs at large average NDDs, making the plateaus in Fig 6B, 6C, 6D and 6E larger for identical neurons.

### Effect of plasticity mechanisms on transition to synchronized states

#### Networks with *hSP-only*

**Fig 7:**
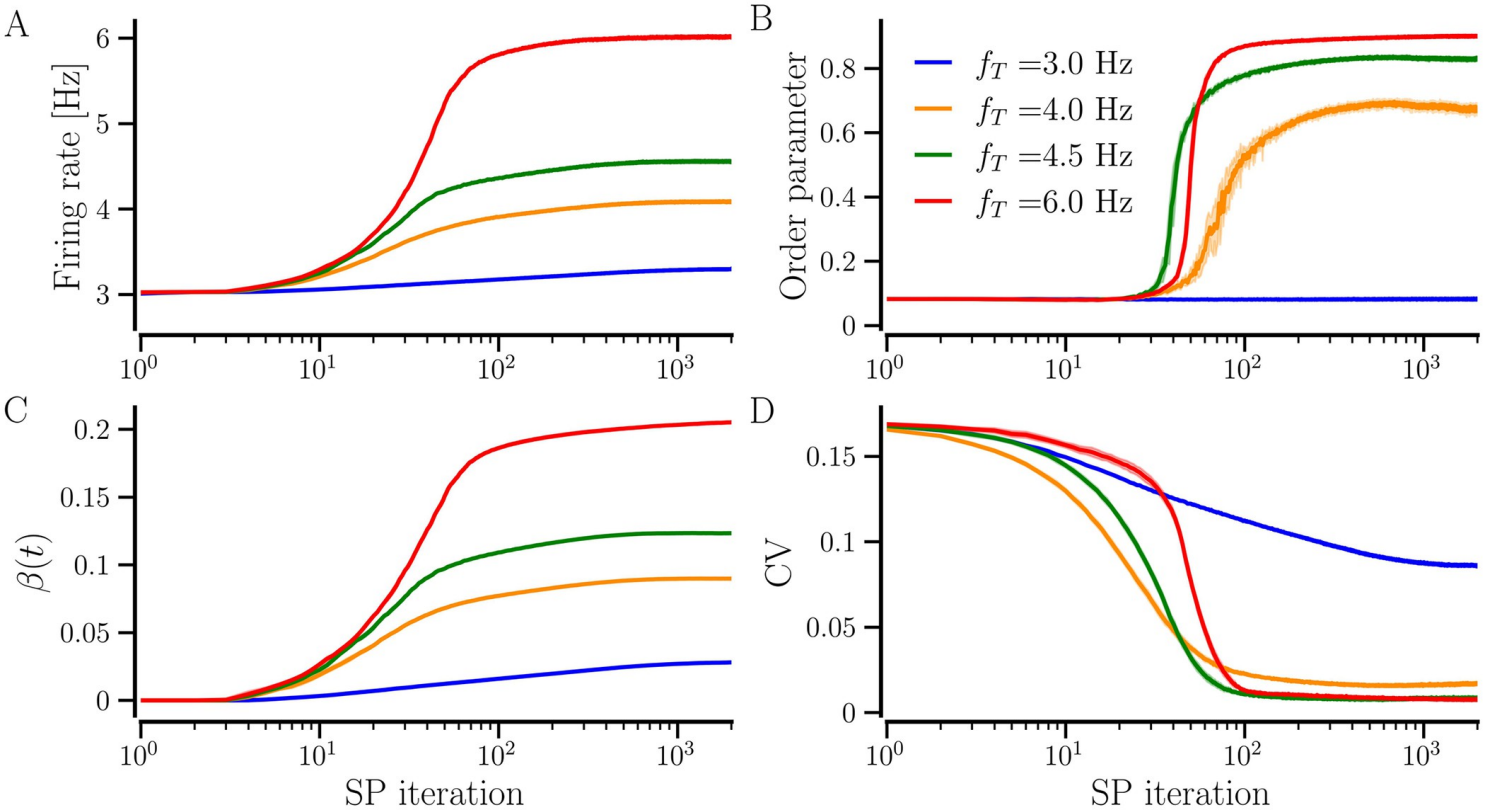
Time evolution of the state measures as the network develops and reaches steady states with *hSP-only*. The network-averaged firing rate (A), the order parameter (B), the network-averaged NDD (C), and the firing rate CV (D). The solid lines show the values averaged over 10 realizations and the shaded area represents the error bars. The diversity parameter is *σ*_*f*_ = 0.5 Hz and other parameters are given in Table 1.

To develop and study the evolution of the network with *hSP-only*, we begin with completely unconnected neurons, i.e., *β*_0_ = 0, and set a target firing rate, *f*_T_. hSP begins to add synaptic contacts with random weights between 0 and 1 in order to bring the firing rate of all the neurons to the target level. The network-averaged firing rate, thus, increases as the average number of contacts, represented by the network-averaged NDD, *β*(*t*), increases over SP iterations and both saturate together to the steady-state values as shown in Fig 7A and 7C. Naturally, a higher average NDD is needed and therefore attained for a higher *f*_T_. A low average NDD may prevent the network from reaching a synchronized state and a high average NDD may allow it to get synchronized. Thus, for smaller *f*_T_ the network ends up in a desynchronized state, e.g., for *f*_T_ = 3 Hz. For a sufficiently large *f*_T_, the network may reach synchrony while for some intermediate *f*_T_ it may reside in a partially synchronized state, e.g., *f*_T_ = 4.5 Hz (or 6 Hz) and 4 Hz, respectively, as shown in Fig 7B. The convergence of firing rates of all neurons in the network to a higher *f*_T_ requires more SP iterations, as indicated by the delayed fall of CV for higher *f*_T_ in Fig 7D. During the transition to the target rate, the neurons have different firing rates, preventing them from getting synchronized. Therefore, the network requires more SP iterations for *F*_*T*_ = 6 Hz than for *F*_*T*_ = 4.5 Hz to get synchronized in Fig 7B. The synchronized states are accompanied by a significantly reduced CV of firing rates as shown in Fig 7D. The network-averaged firing rate overshoots the target when *f*_T_ = 3 Hz because the natural firing rate of the neurons is Gaussian distributed with mean 3 Hz and standard deviation 0.5 Hz. The neurons with natural firing rates below 3 Hz, develop new incoming contacts in order to reach the target firing rate while the ones above 3 Hz do not have any pre-existing contacts to lose, raising the network-averaged NDD and firing rate. The CV of firing rates is determined by the distribution of natural firing rates and the target rate. When the target rate is higher than the natural firing rate of all the neurons (*f*_T_ = 4.5 Hz and 6 Hz in this case), hSP enables all the neurons to attain the target rate and thereby reduces the CV to a minimum. A higher order parameter for *f*_T_ = 6 Hz compared to 4.5 Hz can be attributed to a higher network-averaged NDD. For smaller *f*_T_, the neurons with natural firing rates above *f*_T_ remain unconnected, effectively reducing the order parameter and increasing the CV simultaneously. We observed a similar evolution of network-averaged measures using BvOSP in place of stochastic SP [Fig A in S1 Text].

**Fig 8:**
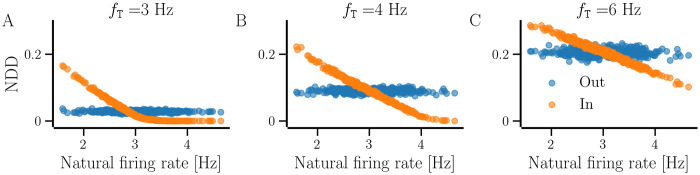
Dependence of in- and out-NDDs on the natural firing rate of the neurons for the network developed with *hSP-only*. The panels A-C correspond to the target firing rates, *f*_T_, specified at the top. All the values are averaged over 10 realizations of random networks. The diversity parameter is *σ*_*f*_ = 0.5 Hz and other parameters are given in Table 1.

Fig 8 shows that in a steady state for a given *f*_T_, the number of presynaptic partners of neurons, represented by in-NDD, decreases with an increase in the natural firing rate below *f*_T_, while the ones above *f*_T_ may not develop incoming contacts. hSP does not directly control the number of postsynaptic partners of neurons and thus, out-NDD displays no dependence on the natural firing rate. The resulting out-NDD is binomially distributed, due to which the spread of out-NDD is larger for larger average NDD, obtained for higher *f*_T_. With BvOSP, we observe a similar dependence of in-NDD on the natural firing rate of the neurons as with our stochastic SP. However, since BvOSP determines both in- and out-NDDs identically based on the natural firing rate of the neurons, the out-NDD shows a similar dependence on the natural firing rate as the in-NDD, shown in Fig B in the S1 Text, unlike that with the stochastic SP.

#### Networks with combinations of structural and weight plasticity

**Fig 9:**
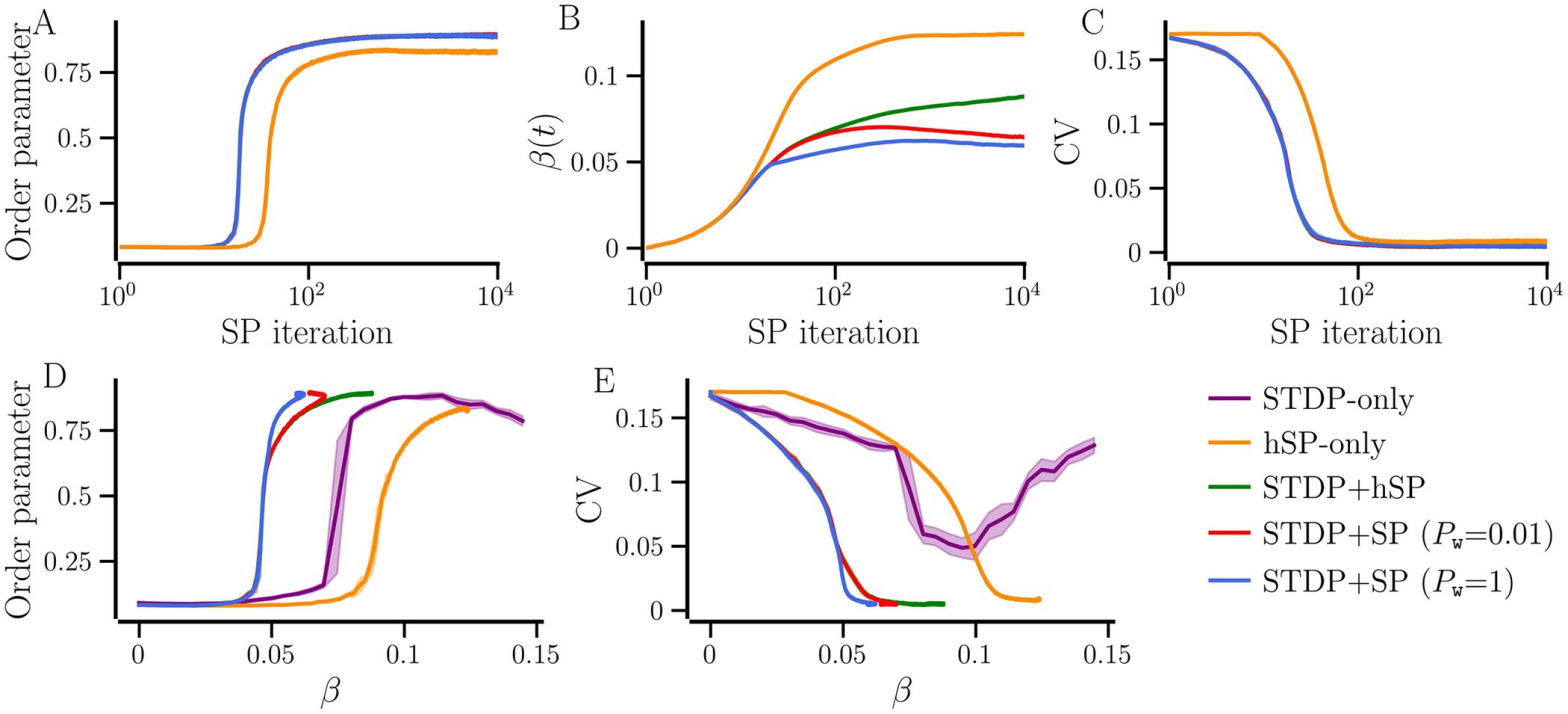
Comparison of network evolution in the presence of different plasticity cases. The change in the order parameter, network-averaged NDD, and firing rate CV with SP iterations (A, B, and C, respectively). Order parameter and the CV of the firing rate versus the network-averaged NDD for five plasticity cases (D and E). The asymmetry parameter, *b*, of STDP, is set to 1, so that the desynchronized steady state is unstable [21]. The solid lines show the values averaged over 10 realizations and the shaded area represents the error bars. Parameters: *P*_*h*_ = 0.01, *σ*_*f*_ = 0.5 Hz.

Here we develop networks using *STDP+hSP* and *STDP+SP* (with *P*_w_ = 0.01 and *P*_w_ = 1) and compare their transition to synchronized states with the network developed using *hSP-only*. With *STDP+hSP* and *STDP+SP*, the new contacts added at each SP iteration (structural update) are given a random initial weight ∈ [0, 0.2] and are subjected to STDP allowing those to get potentiated or depressed. Thus, the neurons can adjust their firing rates by adding and losing contacts besides changing the synaptic weights. The target firing rate is set to 4.5 Hz. This *f*_T_ corresponds to the network-averaged firing rate of a maximally synchronized random network with *STDP-only*, for which the order parameter and the firing rate CV attain their respective maximum and minimum [green curves in Fig 6D and 6E]. Since the target firing rate is higher than the natural firing rates of most of the neurons (mean 3 Hz and standard deviation 0.5 Hz), the initial structural updates involve only the addition of new contacts. The network may get synchronized when a sufficiently large number of contacts have developed with progressing SP iterations. This is indicated by an abrupt increase of the order parameter in Fig 9A and a drop of the firing rate CV in Fig 9C. With *hSP-only*, the network may require a larger number of contacts to get synchronized because some of the strong contacts that are added may not support but rather oppose synchronization. However, for networks with *STDP+hSP* and *STDP+SP*, the synaptic weight change of the contacts may preclude the need to build more contacts, allowing for synchronization at a significantly earlier stage of network evolution and, thus, for smaller average NDD compared to the network with *hSP-only*. Combining Fig 9A and 9B and by excluding the SP iteration axis provides the dependencies of the order parameter on *β* in Fig 9D. Similarly, the dependencies of the firing rate CV on *β* in Fig 9E are obtained by combining Fig 9C and 9B. We add the corresponding curves of the order parameter and the firing rate CV versus average NDD for random networks with *STDP-only* generated for every value of average NDD, as for Fig 6, to compare the transition of networks developed from scratch to random networks with *STDP-only* as well. Fig 9D clearly shows that networks with *STDP+SP* and *STDP+hSP* transition to their synchronized states for significantly lower average NDD (*β* ≈ 0.05) than networks with *STDP-only* (*β* ≈ 0.075) or *hSP-only* (*β* ≈ 0.1). The structural changes continue after the transition to synchronization, and the average NDD converges to a steady state value. In this post-synchronous stage of network evolution, the weight-dependent pruning becomes important. As we demonstrate in the next subsection, the synaptic contacts that become potentiated and promote synchronization tend to survive while the others that become weak get eliminated, thereby optimizing the network structure. Fig 9B shows that as the networks pass the onset of synchronization, the increase in average NDD remains smaller for the networks with *STDP+SP* compared to those with *STDP+hSP* and *hSP-only* and the steady state value of the average NDD decreases with the increase of the probability of weight-dependent pruning, *P*_w_. This is further demonstrated in Fig 9D and 9E, where the curves of order parameter and CV versus *β* cease to change at smaller values of *β* for networks with *STDP+SP*. Importantly, Fig 9E shows that the plasticity of network structure results in synchronized states with much smaller values of the firing rate CV, indicating stronger frequency locking.

In summary, the transition to synchronized states in the presence of any combination of structural and weight plasticity with any form of SP may occur with significantly sparser networks compared to random networks with *STDP-only* or those generated using *hSP-only*. Additionally, hSP and SP that includes hSP and weight-dependent pruning reduce the firing rate mismatch of the neurons, characterized by smaller values of the firing rate CV.

### Effect of including SP on steady synchronized random networks with STDP

#### Enhancement of synchronization

**Fig 10:**
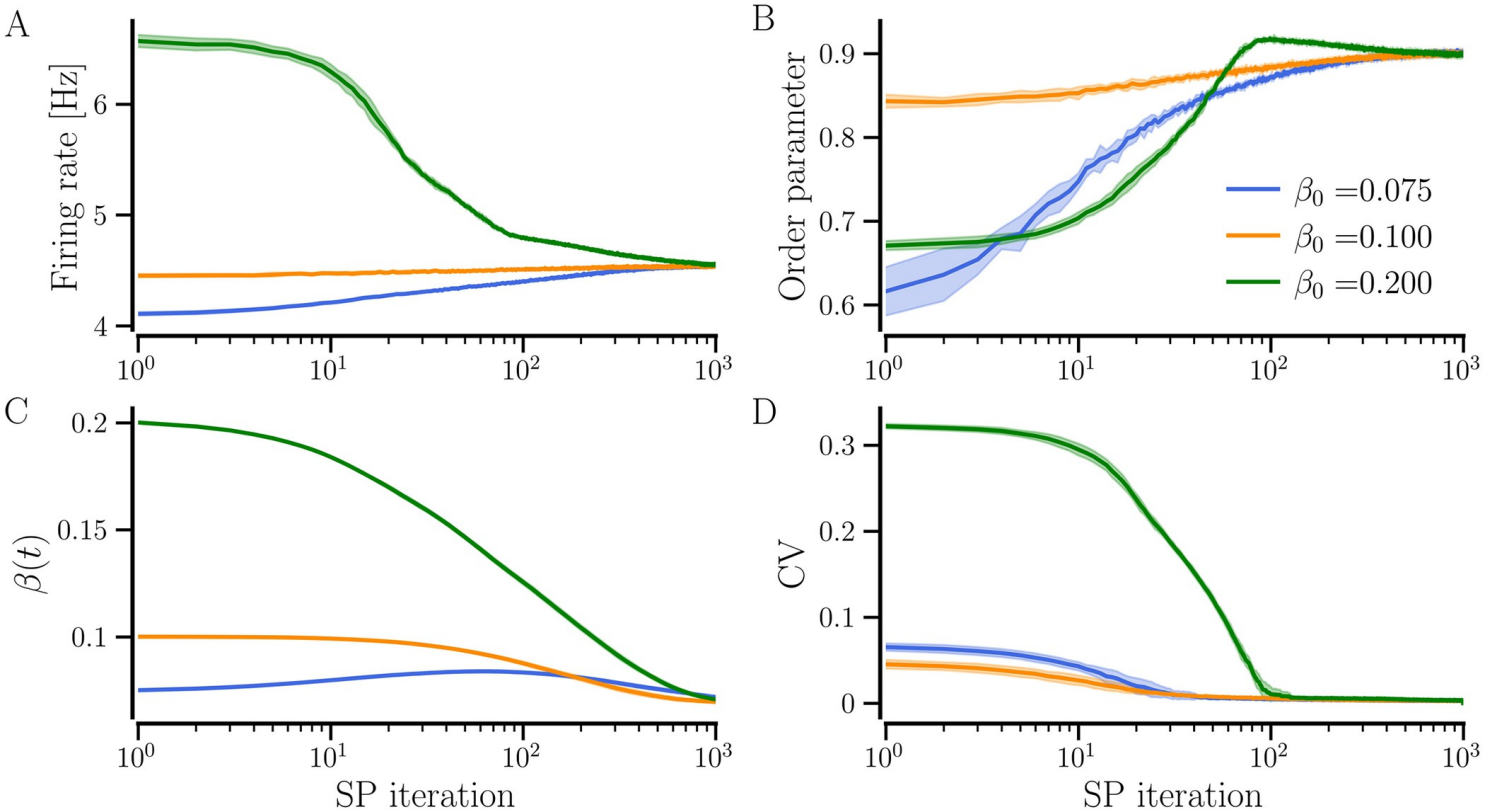
Evolution of network-averaged measures with SP iterations. Evolution of the initially random synchronized networks that progress through SP iterations using STDP+SP are shown. The firing rate is shown in A, the order parameter in B, the average NDD in C, and the CV of firing rates in D. Colors correspond to the three initial values of average NDD (see legend). The solid lines show the values averaged over 10 realizations and the shaded area represents the error bars. Parameters: 〈*W*〉(0) = 0.95, *σ*_*f*_ = 0.5 Hz, and *P*_w_ = 0.01. Other parameters are specified in Table 1.

In our model, synchronized random networks with *STDP-only* tend to have a higher CV of firing rates than the networks that evolve with any plasticity scheme that includes any form of SP [see Fig 9E]. We investigate the effect of taking such synchronized random networks with *STDP-only* through SP iterations, as described in *‘Parameters and implementation’* section. In the presence of SP, neurons tend to become identical as their firing rates converge to the given target rate by adding or losing synaptic contacts, reflected in the CV of firing rates. This may enable the neurons to get more strongly synchronized. We consider synchronized random networks with diversity parameter, *σ*_*f*_ = 0.5 Hz, and set the target firing rate *f*_T_ = 4.5 Hz as above. Fig 10 shows the evolution of the network-averaged measures for three values of initial average NDD, *β*_0_ = 0.075, 0.1, and 0.2, as the networks progress through structural updates with *STDP+SP* (*P*_w_ = 0.01). With SP iterations, the firing rate evolves towards the *f*_T_, a higher degree of synchrony as indicated by larger values of the order parameter, and a smaller value of the firing rate CV in Fig 10A, 10B and 10D, respectively, for all initial average NDDs. The average NDDs of the networks continue to adjust until the target rate is achieved, as shown in Fig 10C. Since *f*_T_ and the probabilities of addition and pruning are the same for all three initial average NDDs, the networks settle with the same average NDD in the steady state, although the underlying network structure may differ. We obtain similar results using BvOSP in place of stochastic SP even though it allows for multiple synaptic contacts between each pair of pre- and post-synaptic partners; the results are presented in Fig C in S1 Text.

**Fig 11:**
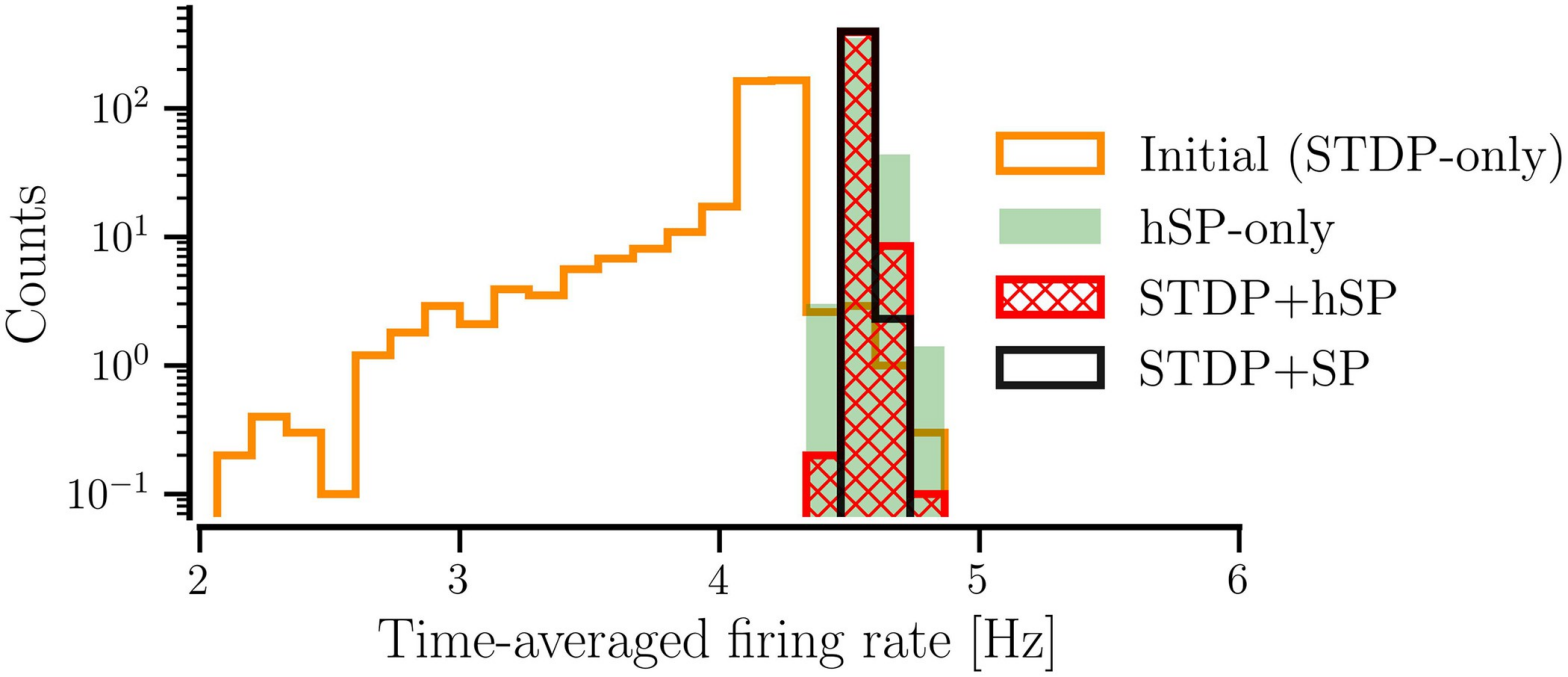
Distributions of steady-state firing rates of all *N* = 400 neurons in the network for different plasticity cases. The initial distribution of firing rates is that of the synchronized random network with *STDP-only*. The networks were initialized with 〈*W*〉_0_ = 0.95 and *β*_0_ = 0.075 for all cases but *hSP-only*, *σ*_*f*_ = 0.5 Hz for all cases, and *P*_w_ = 0.01 for the network with *STDP+SP*. The values are averaged over 10 realizations of the network for each plasticity case.

Fig 11 shows the steady state distribution of the firing rates of the original synchronized random network with *STDP-only* (labeled as initial) and the distributions in the steady states after the network evolved with either *STDP+hSP* or *STDP+SP* (*P*_w_ = 0.01). The distribution for the synchronized random network with *STDP-only* is broad and has a peak near its mean value of ≈ 4.2 Hz. In the steady states with both *STDP+hSP* and *STDP+SP*, the distributions become narrow with peaks at the corresponding mean value of 4.5 Hz (same as *f*_T_), indicating a significantly lower firing rate mismatch or CV compared to the original network with *STDP-only*. We added the distribution for the network constructed from scratch using *hSP-only* to show the reduction in firing rate mismatch in the presence of hSP alone.

**Fig 12:**
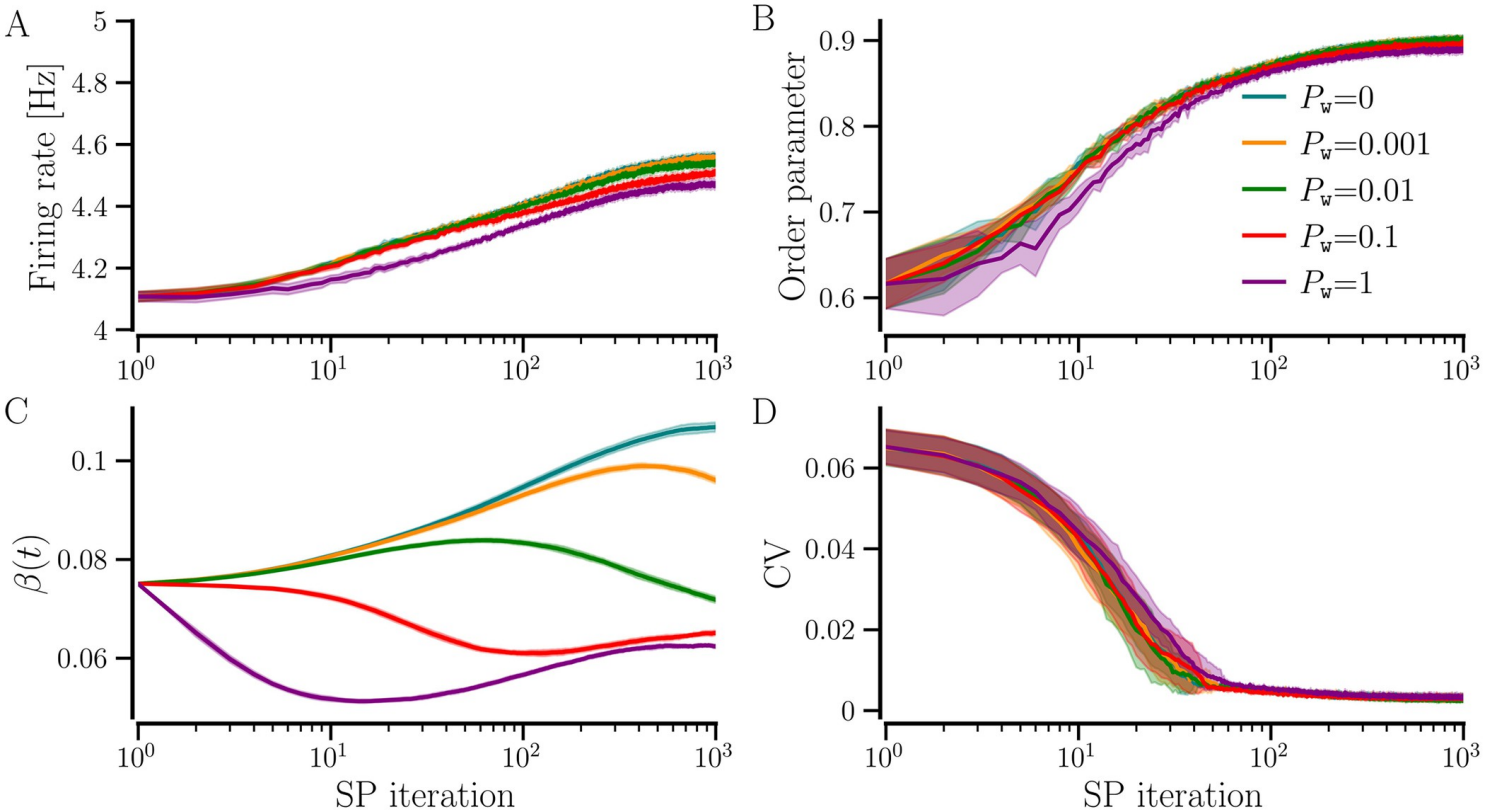
The dependence of network dynamics on the maximal probability of weight-dependent pruning, *P*_w_. Evolution of network-averaged measures as the initially random synchronized network progresses through SP iterations using STDP+SP for the indicated values of *P*_w_. *STDP+SP* becomes *STDP+hSP* for *P*_w_ = 0. The firing rate is shown in A, the order parameter in B, the average NDD in C, and the CV of firing rates in D. The solid lines show the values averaged over 10 realizations and the shaded area represents the error bars. Parameters: *β*_0_ = 0.075, 〈*W*_0_〉 = 0.95, *f*_T_ = 4.5 Hz, and *σ*_*f*_ = 0.5 Hz. Other parameters are specified in Table 1.

To demonstrate the enhancement of synchronization when an initially random network with *STDP-only* progresses through SP iterations for both *STDP+hSP* and *STDP+SP*, we consider a synchronized random network with average NDD, *β* = 0.075, and set the target firing rate *f*_T_ = 4.5 Hz as above. We use different values of the maximal probability of weight-dependent pruning, *P*_w_, while keeping the probability of homeostatic addition and pruning constant, *P*_*h*_ = 0.01. Fig 12B shows that the order parameter increases with SP iterations starting from lower values corresponding to the different realizations of the initial synchronized random network with *STDP-only* for all values of *P*_w_, demonstrating the enhancement of synchronization regardless of *P*_w_. Interestingly, the order parameter, the network-averaged firing rate, and the CV of firing rate evolve similarly and converge to almost equal steady-state values for all considered values of *P*_w_, as shown in Fig 12. However, the evolution of average NDD depends on *P*_w_ until the target firing rate is attained by the network, and the steady-state average NDD decreases with the increase in *P*_w_. It is observed because when *P*_w_ ≠ 0, the weaker contacts get pruned. A higher *P*_w_ causes the pruning of a larger number of weak contacts. The weight-dependent pruning is absent for *P*_w_ = 0 (i.e., *STDP+hSP*), thus the average NDD remains higher than that for *P*_w_ ≠ 0. This reduction in average NDD in the synchronized state due to weight-dependent pruning while the network order parameter remains high points to optimization of the network structure such that the contacts that support or promote synchrony are retained while the others that may not contribute to synchronization are eliminated. The convergence of the network-averaged firing rate to the target firing rate depends on the steepness of the logistic curves of the hSP rule (see Fig 1), controlled by the parameter Δ*f*. Less steep logistic curves used in this study may result in slight overshooting (for small values of *P*_*w*_) or undershooting (for *P*_*w*_ close to 1) of the network-averaged firing rate relative to the target firing rate. Steeper logistic curves result in almost exact convergence of the network-averaged firing rate. Replacing the stochastic SP method with BvOSP gives similar results (see Fig D in the S1 Text).

#### Statistics of synaptic contacts in a synchronized state

**Fig 13:**
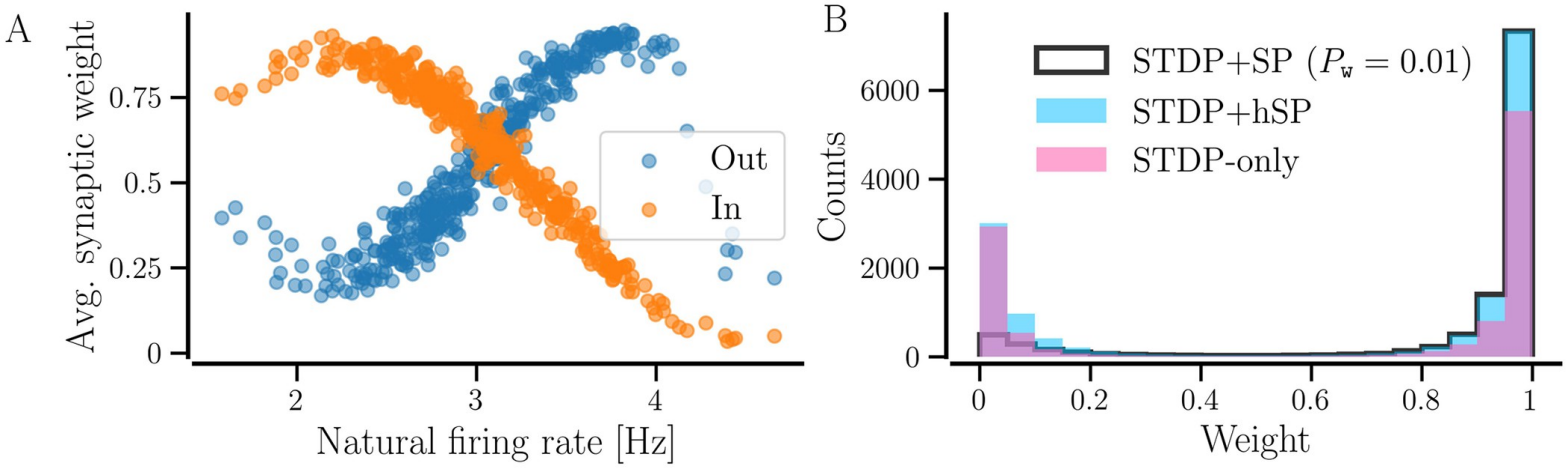
Average synaptic weight depends on the natural firing rate of neurons and weight distributions differ for different plasticity cases. A shows the dependence of average incoming and outgoing synaptic weight on the natural firing rate of neurons for a network with *STDP-only*. B shows the distribution of the synaptic weights of individual contacts for three cases of plasticity: *STDP-only*, *STDP+hSP*, and *STDP+SP* (*P*_w_ = 0.01). The values are averaged over 10 realizations of random networks. Other parameters are the same as in Fig 12.

During the transition to a synchronized state, the firing rates of the neurons converge to steady-state values close to the network average. In order to reach the steady-state values, the incoming contacts of the neurons with lower natural firing rates get potentiated and those of the neurons with a higher natural firing rate get depressed on average due to STDP. At the same time, the outgoing contacts of neurons with higher (lower) firing rates tend to get potentiated (depressed) on average, resulting in a decrease in the average incoming synaptic weight of neurons, and an increase in the outgoing weight is observed with an increase in the natural firing rate, as shown in Fig 13A. Correspondingly, the distribution of the weights of individual synaptic contacts in Fig 13B[pink color] shows two peaks at 0 and 1 in the steady synchronized state of a random network with *STDP-only*.

When a random network with *STDP-only* goes through structural updates with *STDP+SP* (*P*_w_ ≠ 0), the weaker contacts get pruned preferentially, while the existing and the newly added contacts that get potentiated continue to dwell and accumulate over time to raise the firing rates of the neurons to the given target rate. For that reason, the peak in the weight distribution at 0 in Fig 13B reduces considerably and the peak at 1 becomes larger than that for the synchronized random network with *STDP-only*. When *P*_w_ = 0, i.e., for *STDP+hSP*, both strong and weak contacts may survive due to the absence of preferential pruning of weaker contacts and accumulate over time until the target firing rate is achieved, which results in peaks at 1 and 0 in the synaptic weight distribution in Fig 13B. We observe the same using BvOSP in place of stochastic SP, as shown in Fig E in S1 Text.

**Fig 14:**
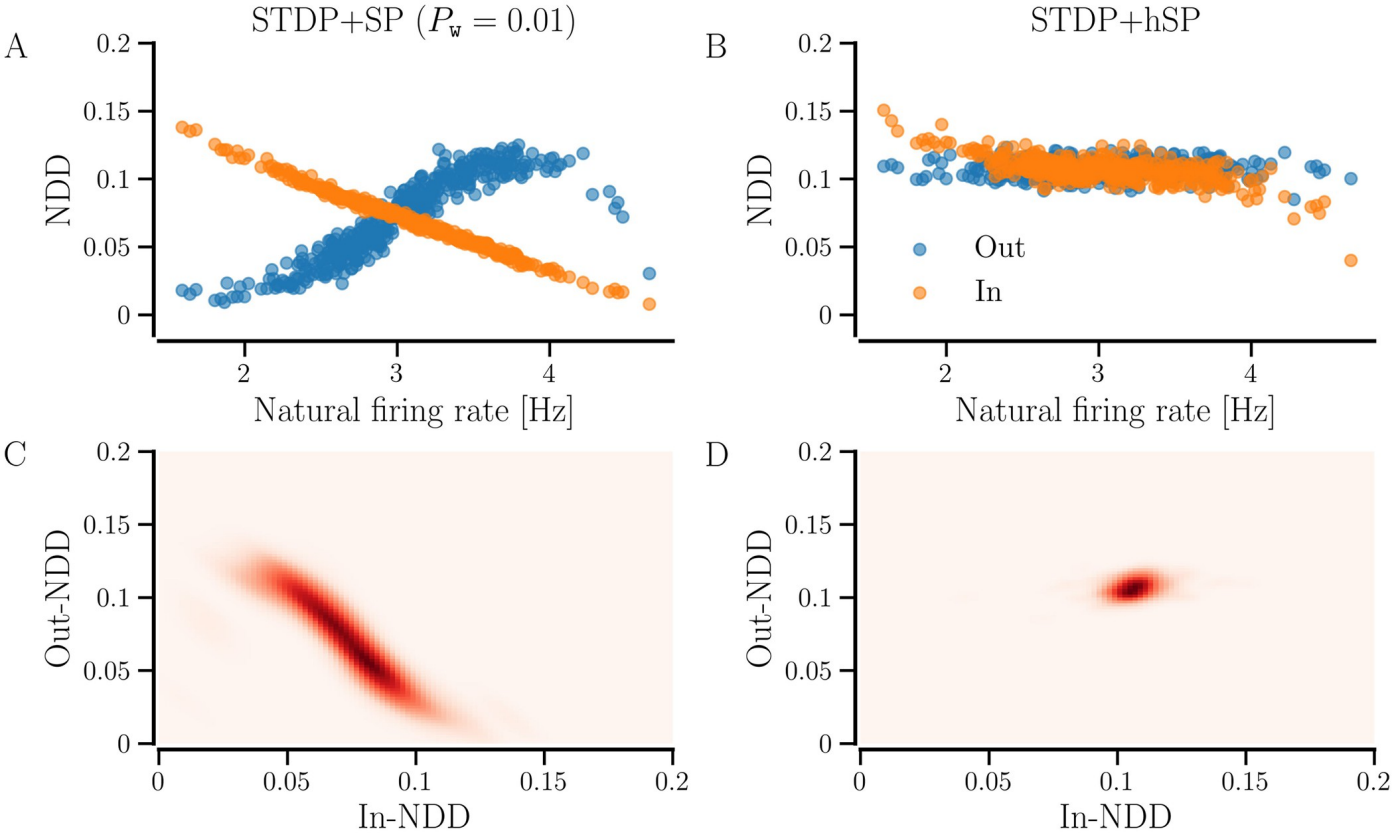
in- and out- NDDs depend on the natural firing rates of the neurons. A and B show the dependence of in- and out- NDDs of networks with *STDP+SP* (*P*_w_ = 0.01) and *STDP+hSP* on the natural firing rates of the neurons in the steady synchronized states, and C and D show their joint probability distribution, *P*(in- NDD, out- NDD). The values are averaged over 10 realizations of initial random networks. Other parameters are the same as in Fig 12.

Owing to the higher (lower) average incoming (outgoing) synaptic weight, the neurons with lower natural firing rates possess more incoming contacts and fewer outgoing contacts when *P*_w_ ≠ 0, as indicated by Fig 14A, since the weaker contacts get pruned. The opposite holds for the neurons with higher natural firing rates. The contrasting dependence of in- and out- NDDs on the natural firing rate results in a negative correlation between those as implicated by their joint probability in Fig 14C. When *P*_w_ = 0, the NDDs do not follow the dependence of synaptic weight on firing rate since weaker contacts are not preferentially pruned. Nevertheless, the neurons with lower natural firing rates require more contacts to reach the target firing rate, whereas those with higher rates require less. Thus, we see a minor dependence of in-NDD on the natural firing rate and none for out-NDD in Fig 14B. The joint probability distribution of in- and out- NDDs in Fig 14D shows a positive correlation between the two, which arises due to the existence of neurons on the boundary of the network that develop fewer incoming and outgoing contacts due to the distance-dependent contact formation. In contrast, the ones away from the boundary can develop a higher number of both. The neurons on the network boundary that possess lower natural firing rates build fewer but stronger contacts during synchronization. Replacing the stochastic SP with BvOSP giver similar results for STDP+hSP but different for STDP+SP, presented in Fig F in S1 Text.

**Fig 15:**
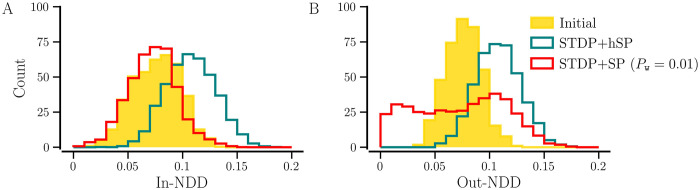
Marginal distributions of in- and out-NDDs for the networks with *STDP+hSP* and *STDP+SP* (*P*_w_ = 0.01) in steady synchronized state. A shows the in-NDD distributions and B shows the out-NDD distributions. The filled histogram shows initial degree distributions with *β*_0_ = 0.075. The values are averaged over 10 realizations of initial random networks. Other parameters are the same as in Fig 12.

Fig 15 compares NDD distributions in steady synchronized states for *STDP+hSP* and *STDP+SP*, i.e., *P*_w_ = 0 and *P*_w_ = 0.01, respectively. For *P*_w_ = 0, both in- and out-NDD distributions have a similar shape and possess the same mode. The accumulation of weaker contacts due to the absence of their preferential pruning results in a shift of the in- and out-NDDs to higher values than the initial distributions. On the contrary, when weight-dependent pruning is on, significantly different distributions of in- and out-NDDs arise. The in-NDD distribution is shifted towards smaller values compared to the initial because of the pruning of weaker contacts. The out-NDD distribution becomes broader and uniform at small values, indicating that neurons possess a wider range of degrees and many have a small number of outgoing contacts. We observe the same using BvOSP in place of stochastic SP, as shown in Fig G in S1 Text.

**Fig 16:**
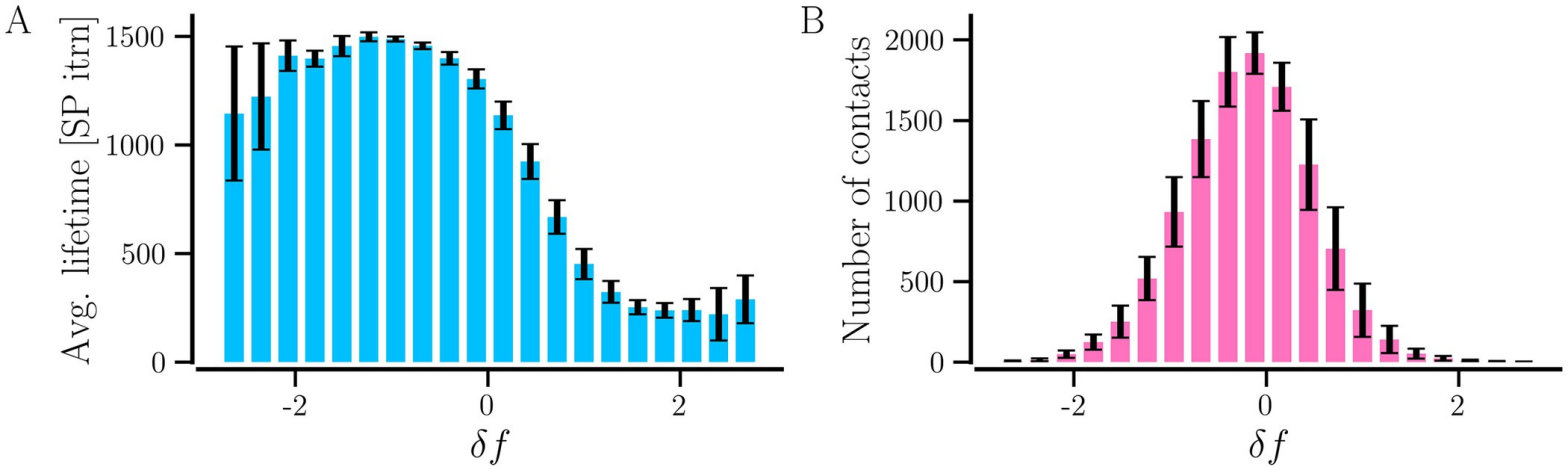
The survival of synaptic contacts is subject to the difference between the firing rates of the neurons connected by them. The dependence of the average lifetime of the synaptic contacts (A) and the number of contacts (B) in the steady synchronized state of the network with *STDP+SP* on the difference between the firing rates of the post- and presynaptic neurons, *δf*. *δf* > 0 when the postsynaptic neuron has a higher firing rate than the presynaptic and *δf* < 0 when the opposite holds. The values are averaged over 10 realizations of initial random networks and the error bars show the corresponding standard deviation. Parameters are *P*_w_ = 0.01, 〈*W*〉 = 0.95, *σ*_*f*_ = 0.5 Hz, and *β*(0) = 0.075. *P*_w_ = 0.1 and 1 give similar results.

The dependence of the average incoming and outgoing synaptic weight of the neurons on their natural firing rate can be attributed to the potentiation of contacts going from neurons with a higher firing rate to those with a lower firing rate and the depression of the ones going the opposite way. This phenomenon is evident from Fig 16A, which shows the dependence of the average lifetime of the contacts on the difference between the natural firing rates of the paired neurons, *δf* = *f*_*i*_ − *f*_*j*_, in a synchronized network in the presence of weight-dependent pruning, i.e., *STDP+SP* (*P*_w_ = 0.01). Here *f*_*i*_ is the natural firing rate of the postsynaptic neuron and *f*_*j*_ is that of the presynaptic neuron. The average lifetime of contacts that appear between a given pair of neurons is calculated after the network settles in a steady synchronized state with 500 SP iterations. When the postsynaptic neurons fire at a higher rate than the presynaptic (*δf* > 0), most contacts become short-lived owing to their depression. In contrast, the contacts between neurons with *δf* < 0, tend to live for longer because of potentiation. As a result, more contacts exist between neurons with *δf* < 0 in the steady synchronized state, as indicated by the distribution of *δf* for connected pairs of neurons in Fig 16B. The peak in distribution close to 0 indicates that neurons with similar natural firing rates are more likely to remain connected in a synchronized state.

**Fig 17:**
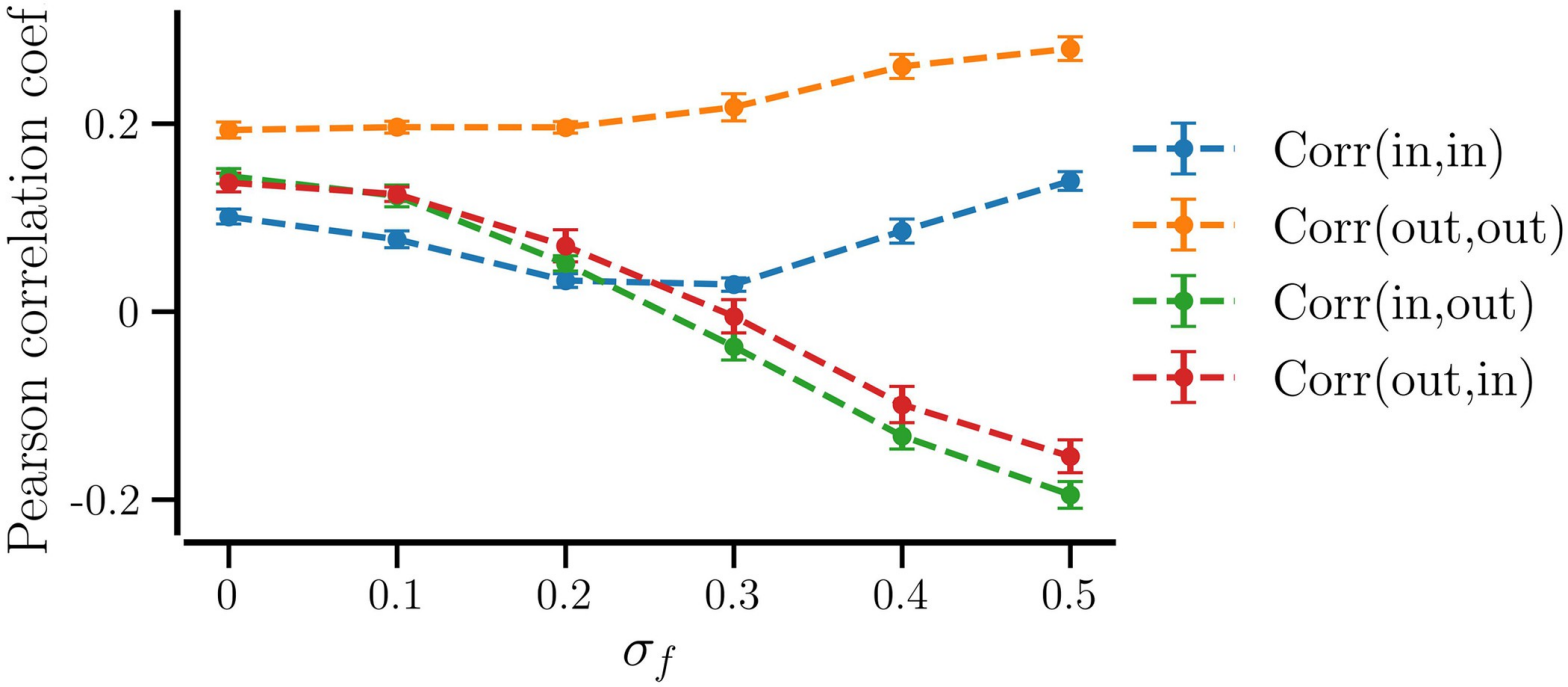
Network degree-(dis)assortativity depends on the diversity parameter, *σ*_*f*_. The Pearson correlation calculated between the NDDs of the pre- and post-synaptic neurons, labeled as (NDD-type of pre, NDD-type of post) is shown. The values are averaged over ten realizations of initial random networks and the error bars show the standard deviation for each value of *σ*_*f*_. Parameters: *P*_w_ = 0.01, *β*_0_ = 0.075, and 〈*W*〉 = 0.95.

Next, we examine the emergence of correlation between the node degrees of connected neurons in a synchronized network with *STDP+SP* (*P*_w_ = 0.01). The standard measure of node degree correlations is the Pearson correlation coefficient (Eq 18), which also serves as a degree assortativity measure for complex networks [56, 87, 88]. For a directed graph, this measure gives four values assessing correlations between (in-in), (out-out), (in-out), and (out-in) node degrees [88, 90], where the first degree-type corresponds to the presynaptic neuron and the second one corresponds to its postsynaptic partner. Fig 17 shows the Pearson correlation coefficient values versus the diversity parameter, *σ*_*f*_. The correlation coefficient is averaged over ten realizations of networks for each value of *σ*_*f*_. A positive (negative) correlation value implies that the network is assortative (disassortative). We observe that for identical neurons or smaller diversity (*σ*_*f*_ < 0.2), the networks become assortative for all degree types of the pre- and post-synaptic neurons. For larger diversity (*σ*_*f*_ > 0.2), a mixture of assortativity and disassortativity emerges as reported for other networks [89]. On the one hand, the (in-in) and (out-out) correlations are positive and increase with an increase in *σ*_*f*_, implying that the networks are in-assortative and out-assortative [90] and the networks with a higher *σ*_*f*_ are more assortative. On the other hand, the negative (in-out) and (out-in) correlations and their decrease to more negative values with an increase in *σ*_*f*_ imply that in that respect, the networks become increasingly disassortative [89].

The emergence of non-zero node-degree correlations can be attributed to two factors: first, the spatial arrangement of neurons on a square lattice accompanied by distance-dependent formation of contacts, and second, the existence of more synaptic contacts between neurons with similar natural firing rates in a synchronized state, as suggested by the peak around 0 in Fig 16B. The boundary effect arises due to the availability of fewer neurons close to the network edges, letting the neurons on the edges build fewer incoming and outgoing contacts. This can be countered by firing-rate-dependent incoming contact formation but it prevails for the outgoing contacts as those are not added based on the firing rate of the presynaptic neurons. Consequently, the out-degree of neurons on the edges is smaller and that of neurons away from the edges is larger; combined with the likelihood of forming contacts with more nearby neurons, it results in higher positive values of (out-out) correlation regardless of the diversity parameter. The firing-rate-dependent incoming contact formation makes the neurons for small *σ*_*f*_ < 0.2 have similar degrees, resulting in positive (in-in), (in-out), and (out-in) correlations. However, the degrees show increasing dependence on the natural firing rates as *σ*_*f*_ increases due to the weight-dependent pruning. Thus, when neurons with similar firing rates get connected, they are more likely to have similar degrees for a large *σ*_*f*_, resulting in a higher (in-in) correlation. At intermediate values of *σ*_*f*_, the dependence of both in- and out-NDDs on natural firing rate is weaker than that for a larger *σ*_*f*_, resulting in a lower (in-in) correlation. Since the in- and out- degrees depend oppositely on the natural firing rates [Fig 14A], the (in-out) and (out-in) correlations become increasingly negative for larger *σ*_*f*_. Without weight-dependent pruning (*P*_w_ = 0), the networks show no significant node degree correlations, i.e., the Pearson correlation coefficient is not significantly different from 0.

### Desynchronized states with a combination of structural and weight plasticity

Steady synchronized states are obtained when the initial average NDD and synaptic weight are sufficiently high [cf. Figs 3 and 6]. In our model, a steady synchronized state is reached when the network-averaged firing rate reaches *f*_T_, bringing the average NDD to its steady value. A desynchronized state of a random network with *STDP-only* is obtained when the initial synaptic weights are small. In desynchronized states, the neurons remain weakly coupled due to the persistent depression of most of the synaptic weights. Consequently, the network-averaged firing rate may shift to larger values than the average natural firing rate, *f*_0_, but may not reach *f*_T_ if it is much higher than *f*_0_. If a desynchronized random network with *STDP-only* undergoes SP iterations as described in *‘Parameters and implementation’* section, the initial structural updates only involve the addition process because of lower firing rates of the neurons. The neurons may continue to develop contacts while never reaching *f*_T_ until the network reaches all-to-all connectivity. The pruning process slows down the increase in average NDD and may halt it by balancing the number of added contacts. Consequently, a steady desynchronized state of a network with *STDP+SP* or *STDP+hSP* is reached when the pruning process balances the addition, bringing the average NDD to a steady value. The rates at which contacts are added and pruned may, therefore, have a significant impact on the network density in the steady desynchronized state.

**Fig 18:**
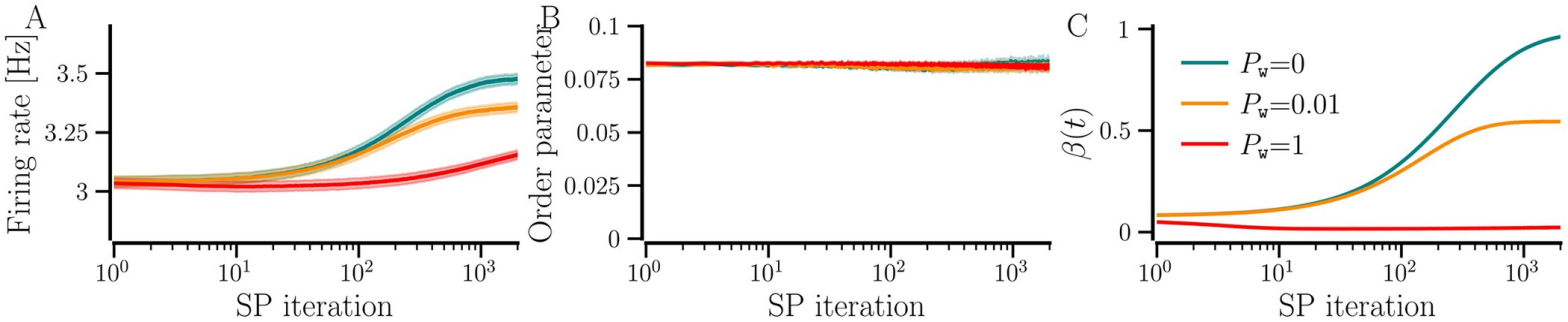
Evolution of network-averaged measures in the desynchronized state. Networks with *STDP+hSP* (*P*_w_ = 0) and *STDP+SP* (*P*_w_ ≠ 0) for the indicated values of *P*_w_ are shown. A shows the firing rate, B shows the order parameter, and C shows the average NDD. The solid lines show the values averaged over 10 realizations and the shaded area represents the error bars. Parameters: *β*_0_ = 0.075, 〈*W*_0_〉 = 0.05, and *σ*_*f*_ = 0.5 Hz. Other parameters are specified in Table 1.

We study the effect of the maximal probability of weight-dependent pruning, *P*_w_, on the steady-state average NDD of the network in a desynchronized state. SP updates were implemented until the average NDD converged with the relative accuracy of 0.1%. We set *f*_T_ = 4.5 Hz as for the synchronized networks in the above sections so that the target firing rate is higher than the natural firing rates of all the neurons in the network. Fig 18A shows that the network-averaged firing rate may increase in the desynchronized state depending on *P*_w_ but fail to reach *f*_T_ since it is much higher than *f*_0_ = 3 Hz. Additionally, the homeostatic pruning remains negligible. Therefore, Fig 18C shows that in the absence of weight-dependent pruning, *P*_w_ = 0 (*STDP+hSP*), the neurons continue to build contacts and the network becomes densely connected. With weight-dependent pruning, i.e., *P*_w_ ≠ 0, the network loses weak contacts, reducing the steady-state average NDD. This effect increases with increasing *P*_w_.

**Fig 19:**
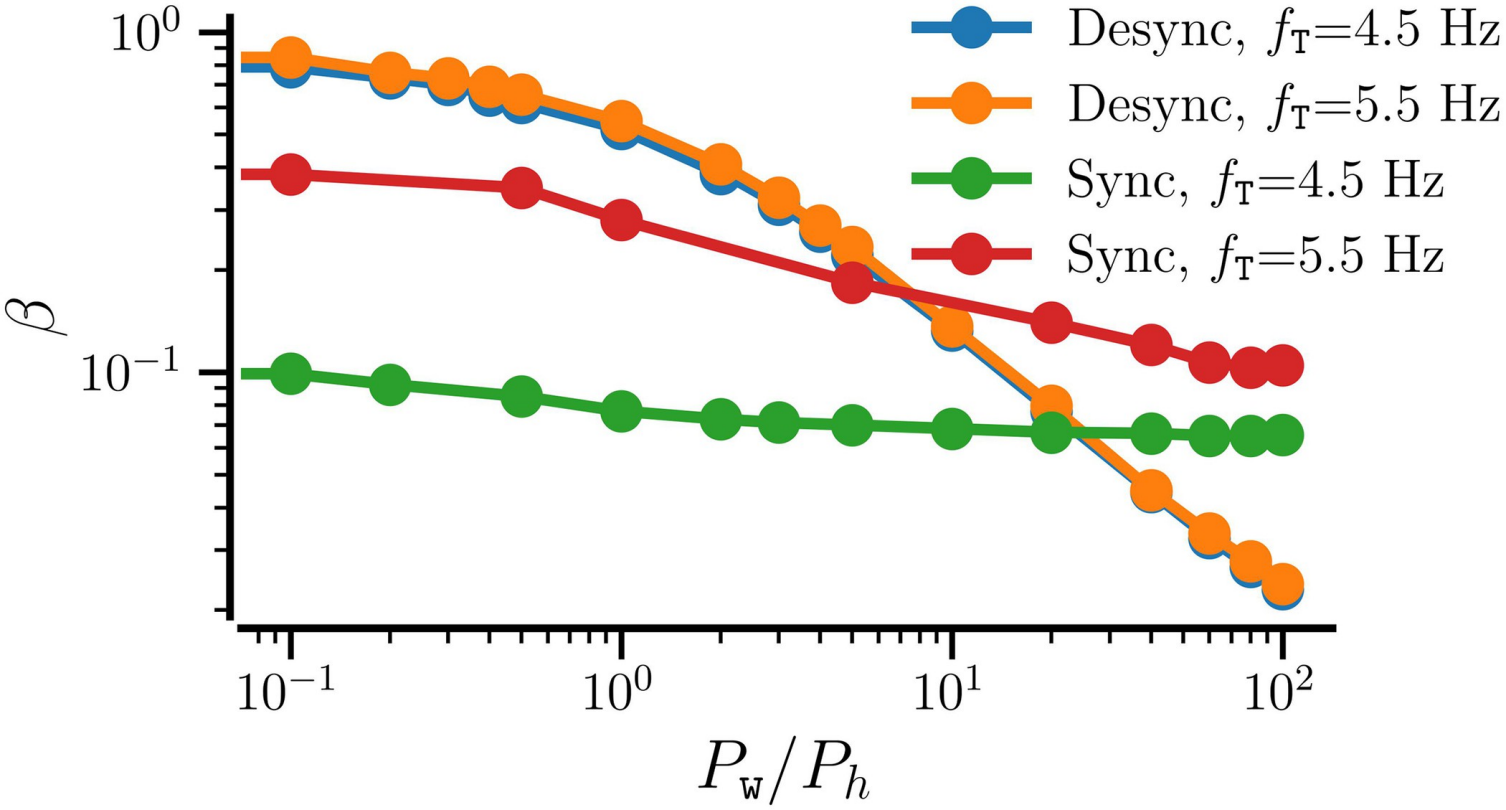
Steady-state average NDD depends on the probabilities of homeostatic SP and weight-dependent pruning. The value of the steady-state average NDD, *β*, versus the ratio of the maximal probability of weight-dependent pruning, *P*_w_, to the probability of homeostatic addition and pruning, *P*_*h*_, for the indicated values of the target firing rate in desynchronized and synchronized states. The initial value of average NDD is *β*_0_ = 0.075; the initial average weight is 〈*W*_0_〉 = 0.05 for desynchronized states, and 〈*W*_0_〉 = 0.8 for synchronized states.

Fig 19 shows the decrease in steady state values of the average NDD in the desynchronized state with an increase in *P*_w_ when *P*_*h*_ = 0.01 for *f*_T_ = 4.5 and 5.5 Hz. We also present the average NDD in a steady synchronized state of the network for the same values of *f*_T_ in Fig 19 for comparison. For sufficiently high values of *P*_w_/*P*_*h*_, the desynchronized network has a significantly lower average NDD than the synchronized network. For example, for *f*_T_ = 4.5 Hz, *P*_w_ = 1 and *P*_h_ = 0.01, the desynchronized network has *β* ≈ 0.023 whereas the synchronized network has *β* ≈ 0.062. It is consistent with the results reported in our previous study that used a simple phase oscillator model for neurons [53]. On the contrary, when *P*_w_ and *P*_*h*_ are comparable, the desynchronized network can be significantly denser than the synchronized network. For example, for *P*_w_ = *P*_h_ = 0.01, the desynchronized network has *β* ≈ 0.5 whereas the synchronized network has *β* ≈ 0.07, for *f*_T_ = 4.5 Hz.

### Effect of desynchronizing stimulation

The synchronized spiking of neurons in our network may be used as a simple model representing pathological states of certain brain areas in neurological disorders, such as PD [97], epilepsy [98], and tinnitus [99, 100]. A number of stimulation methods have been designed to counter abnormal synchrony, induce long-term desynchronization, and restore normal spiking pattern, for instance, in PD [24, 32, 33].

**Fig 20:**
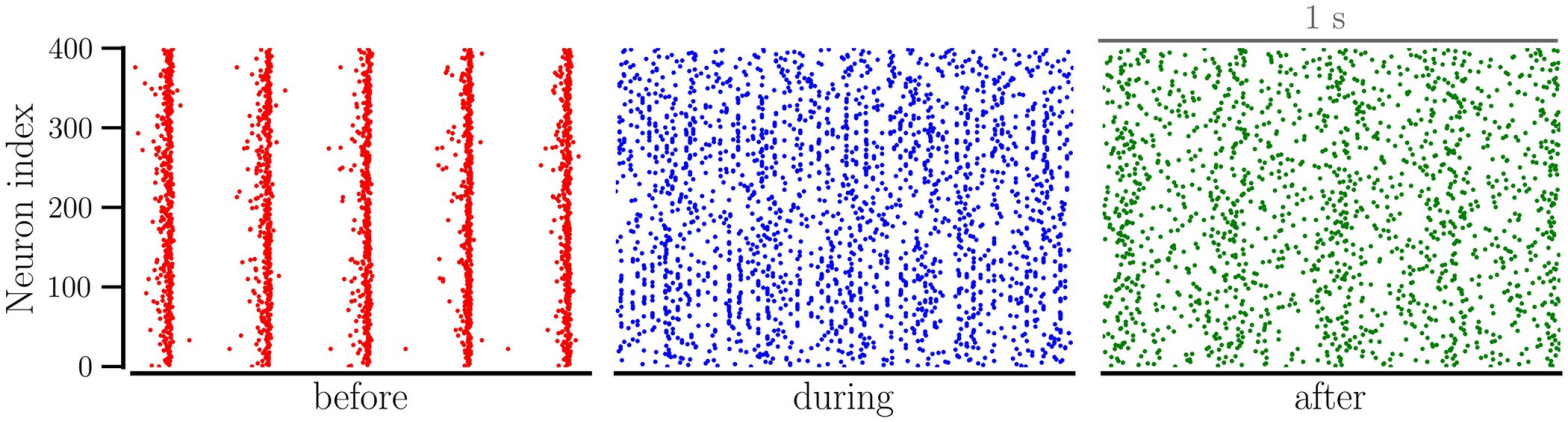
Effect of UMRS stimulation on a synchronized network with *STDP-only*. The raster plot on the left shows a synchronized state, the middle raster is obtained during the desynchronizing stimulation period, and the raster on the right shows the desynchronized state achieved post-stimulation. Stimulation parameters: *a*_*s*_ = 0.4, *F*_*s*_ = 50 Hz, and duration = 2 minutes. Other parameters are specified in Table 1.

The raster plots in Fig 20 exemplify the effect of desynchronizing stimulation, UMRS, on a synchronized network with *STDP-only*. The stimulation disrupts the synchronized firing (middle panel), causing a desynchronization and moving the network into the basin of attraction of a desynchronized state. Accordingly, after removing the stimulation, the desynchrony persists, as shown in the right-most panel.

As demonstrated in previous sections, the type of plasticity affects the spontaneous dynamics of the networks. The synaptic reorganization that occurs when STDP is combined with SP (or hSP) enables the network to become more strongly synchronized than the random network with *STDP-only* (see Figs 9, 10, and 12). To study the effect of UMRS on the synchronized networks, we consider two random networks with *STDP-only*, one with *STDP+SP* (*P*_w_ = 0.01), and one with *STDP+hSP*. For given parameters, *σ*_*f*_ = 0.5 Hz, *P*_w_ = *P*_*h*_ = 0.01, and *f*_T_ = 4.5 Hz, the network with *STDP+SP* settles in a synchronized state with *β* ≈ 0.07 starting from any value of *β*_0_ as seen in Fig 10. However, a random network with *STDP-only* with the same parameters and *β*_0_ = 0.07 does not converge to a synchronized state [Figs 3 and 6]. Thus, we consider a random network with *STDP-only* having *β*_0_ = 0.08 to keep the average NDD of the networks with and without SP very similar and prepare both in a synchronized state. This network, however, has a lower order parameter, 0.7, and network-averaged firing rate, ≈ 4.2 Hz, than the network with *STDP+SP*, with 0.9 and ≈ 4.5 Hz, respectively. Thus, we also consider a random network with *STDP-only* having *β*_0_ = 0.105 with an order parameter 0.86 and network-averaged firing rate 4.5 Hz, close to the network with *STDP+SP*. The network with *STDP+hSP* settles with *β* = 0.106 and the order parameter and average firing rate similar to those of the network with *STDP+SP*. To highlight the role of network structure that emerges in the presence of SP or hSP on the robustness of networks against stimulation, we halt the structural updates during and after stimulation.

**Fig 21:**
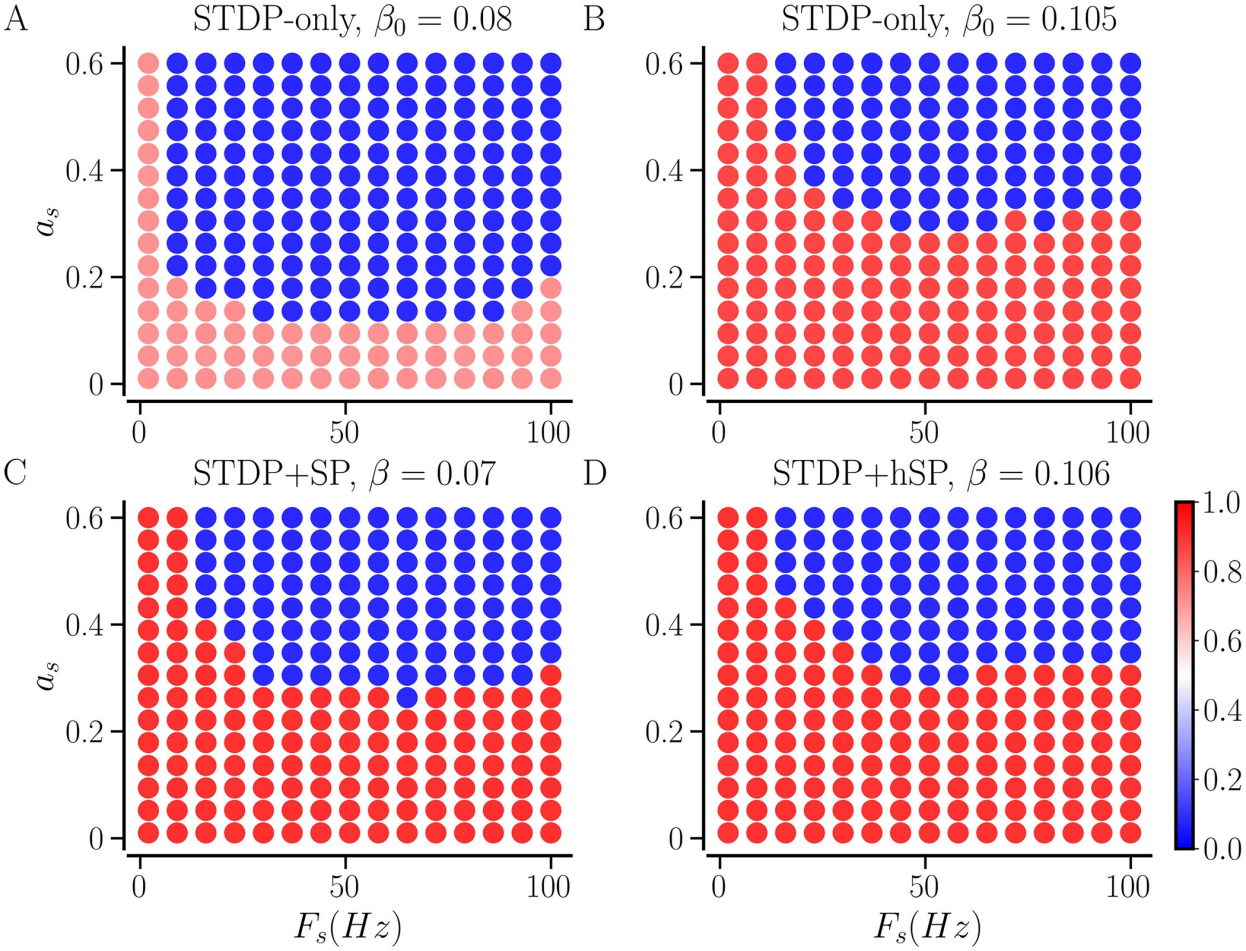
Desynchronization of synchronized networks after stimulation using UMRS. A and B show networks that evolved with *STDP-only*, C shows the network with *STDP+SP* (*P*_w_ = 0.01), and D shows the network with *STDP+hSP* (*P*_w_ = 0). The colors show the order parameter of the networks five hours after a two-minute stimulation window for given combinations of stimulus amplitude and frequency. Red indicates that the network returns to a synchronized state after stimulation, while blue indicates that it settles in a desynchronized state. The stimulus parameters are given in Table 1.

Fig 21 compares the intensity and frequency of stimulus needed to induce desynchronization in the networks considered. The maps show two regions of the combinations of amplitude and frequency of the stimulus current administered for a two-minute window. The blue regions show those combinations that successfully desynchronize the initially synchronized networks, while the red regions show those that do not. The extension of the blue region in Fig 21A to lower values of amplitude and frequency than Fig 21C indicates that when the average NDDs of the network with *STDP-only* and the one with *STDP+SP* are close, the one with *STDP-only* requires much lower amplitude and frequency of stimulus to get desynchronized. The blue and red regions in Fig 21B, 21C and 21D show negligible differences, implying that the network with *STDP-only* as well as the network with *STDP+hSP* require much higher average NDD to be as robust against stimulation as a network that evolved with *STDP+SP*.

**Fig 22:**
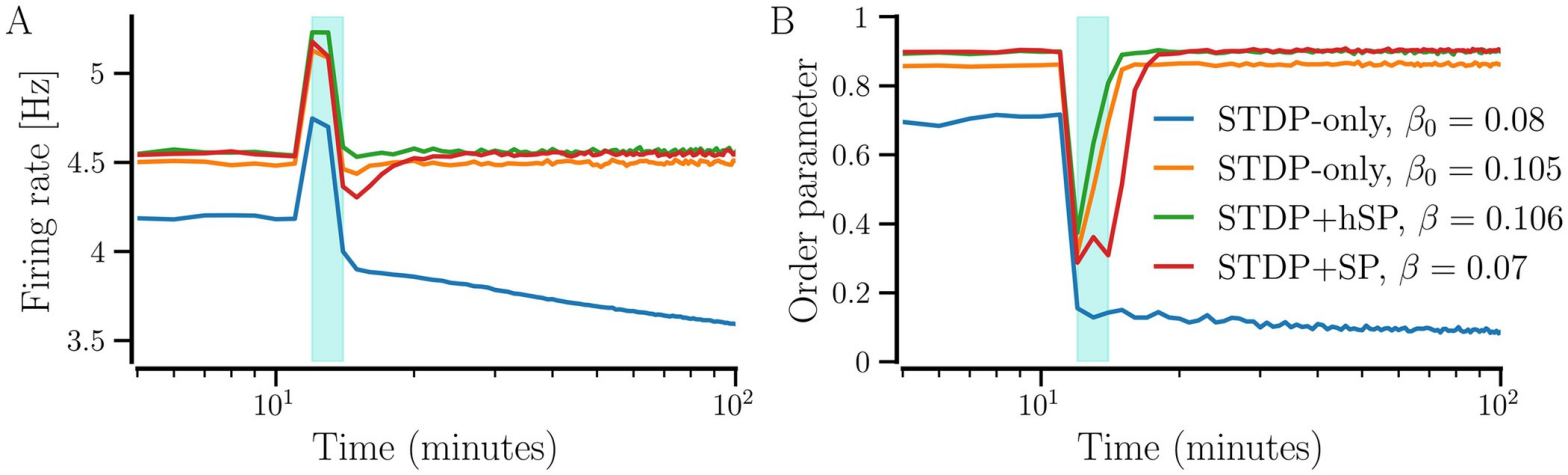
An example of the effect of UMRS stimulus on the synchronized networks with specified plasticity cases. The network-averaged firing rate and the order parameter of the network are shown in A and B, respectively. The shaded area shows the stimulation period. *P*_w_ = 0 for STDP+hSP and *P*_w_ = 0.01 for STDP+SP. Stimulation parameters: *a*_*s*_ = 0.221, *F*_*s*_ = 51 Hz, and duration = 2 minutes. Other parameters are specified in Table 1.

Fig 22 illustrates the effect of UMRS stimulus by showing an example of the time evolution of the network-averaged firing rate and the order parameter of the networks considered. All four synchronized networks initially have higher network-averaged firing rates and order parameters. During stimulation, the firing rate increases while the order parameter decreases for all the networks. However, post-stimulation, only the random network with *STDP-only* and *β*_0_ = 0.08 gets desynchronized as implied by its low order parameter after stimulation, while the other networks relax back to their synchronized states. This is expected since the random network with *STDP-only* with *β*_0_ = 0.08 is initially less strongly synchronized as indicated by its lower order parameter compared to the other networks. The networks with *STDP+SP* and *STDP+hSP*, and the random network with *STDP-only* with *β*_0_ = 0.105 have similar levels of synchrony and thus, respond similarly to the desynchronizing stimulation.

## Discussion

Using model networks of heterogeneous conductance-based integrate-and-fire neurons with adaptive synaptic contacts, we studied how the neuronal activity, synaptic weights, and the network structure co-evolve towards coherent (synchronized) and incoherent (desynchronized) spiking states. The extent of synchronized spiking of neurons within specific brain areas may differentiate pathological from normal conditions. For example, excessive synchronization in the STN and basal ganglia may impair motor function in patients with Parkinson’s disease [29–31]. Conversely, a reduction in synchronized firing of fast-spiking interneurons reduces gamma oscillations in the hippocampus which, in turn, may impair cognitive function in patients with Alzheimer’s disease [37]. The primary circadian pacemaker in mammals, the suprachiasmatic nucleus, relies on its neurons’ interactions and their synchronized activity for normal functioning [101]. Therefore, we emphasized synaptic reorganization arising from the interaction of neurons and the interplay of plasticity mechanisms in synchronized neuronal networks. Additionally, we evaluated the robustness of synchronized networks against external desynchronizing stimulation, which is relevant to the development of therapeutic stimulation methods for Parkinson’s disease.

Networks of integrate-and-fire neurons [21, 23] and other neuron models [33, 53, 64, 102, 103] with synaptic plasticity are known to display multistability. We employed a widely used additive STDP rule that changes the weight of a synaptic contact based on the precise timings of the spikes of the connected neurons [25, 26] and a simple stochastic SP rule that adds and prunes contacts depending on the firing rate of the postsynaptic neurons. We included weight-dependent pruning that selectively eliminates weaker contacts since thin spines engaging in weak contacts are transient in nature while thick spines form stronger contacts that persist for longer times [40]. We found that the SP can remarkably enhance the synchrony level of heterogeneous networks, i.e., networks with non-identical neurons. Weight-dependent pruning allows for enhanced synchrony with a much smaller number of synaptic contacts, which could be a mechanism for optimizing the network structure to reduce energy consumption by preserving only essential and strategically relevant contacts since synaptic signaling consumes a major fraction of the total energy in the brain [104, 105]. We examined properties of the network structure in steady (de)synchronized states by employing tools from network theory and discovered several interesting features that carry implications for network robustness against external stimulation-induced manipulation.

### Stochastic model of structural plasticity

Different implementations of adaptive neuronal networks with synaptic plasticity, both weight and structural plasticity, have been used to study either the properties of emergent network structures in the equilibrium [46, 47, 106] or the resulting synchronization dynamics [20, 50–53]. Synchronization is an emergent phenomenon arising from the interaction of the nodes and the structure of a network. Thus, it is essential to capture the complex interactions between neuronal dynamics and synaptic plasticity during transitions to and in steady (de)synchronized states in plastic neuronal networks. Previously, synchronization was studied using simpler models of SP with phase oscillators that preserved the number of contacts and preferentially connected the oscillators that either had similar average frequencies [51] or were more out-of-phase [50], which led to stronger clustered or global synchrony, respectively. Recently, synchronization was studied with detailed neuron models combined with synaptic weight and SP models [20, 52, 53]. However, these studies either did not capture the interaction of synaptic weights and structural change by excluding weight-dependent pruning [20, 52] or imposed homeostatic constraints on the node degrees rather than employing an average activity-based hSP rule [53]. In the present study, we captured the interactions of neuronal dynamics, synaptic weights, and structural changes while maintaining distinct timescales of the three by employing STDP that connected neuronal spiking and synaptic weight change, hSP that connected average neuronal activity (firing rate) and structural change, and weight-dependent pruning that captured the interaction of synaptic weight and structural dynamics.

A neuroscientifically informed SP model, BvOSP [61], has been successfully used in many studies, for example, to generate networks from unconnected neurons and study their reorganization [49, 61, 83]. Its algorithm, however, is computationally costly to execute and therefore may limit the extent to which the analysis of network dynamics may be conducted. Our stochastic SP method, on the other hand, abstracts from the details of the spine and bouton genesis, unlike BvOSP, and directly builds contacts between neurons, allowing for faster computational implementation. We combined it with STDP-driven weight dynamics, unlike most previous studies that employed BvOSP [49, 82, 84]. Our stochastic SP method bears some similarities with BvOSP but differs in many regards. BvOSP permits multiple synaptic contacts between any given pair of pre- and post-synaptic partners, but in our stochastic SP, for simplicity, we assumed that a single contact accounts for the overall effect of the presynaptic neuron on its postsynaptic partner, although it can easily be extended to include multiple contacts. BvOSP determines the numbers of pre- and post- synaptic partners for all neurons due to the explicit dependence of birth and death of both axonal and dendritic elements on average neuronal activity, whereas the stochastic SP method only determines the number of presynaptic partners based on a neuron’s activity level, not the number of postsynaptic partners. Our model of SP successfully reproduced the results obtained with BvOSP in this study.

### Networks with *STDP-only* may settle in steady states with different levels of synchronization depending on the initial conditions

To understand how each type of plasticity governs the network dynamics, we first studied the synchronized states of homogeneous and heterogeneous networks consisting of identical and non-identical neurons, respectively, with *STDP-only*. In this context, a perfectly synchronized state is defined by the coincident firing of the entire network. Interestingly, the networks attained fully synchronized states for intermediate values of the average node degree and desynchronized states for lower values, while remaining partially synchronized for higher values of the average node degree. Higher values of the average node degree may cause cluster states. It indicates that high connectivity does not necessarily favor the synchronized activity of neuronal networks with *STDP-only*, in agreement with a previous study [107]. This is also in agreement with pre-clinical evidence. For instance, in Parkinsonian mice, an increase in cortico-STN coherence was accompanied by a substantial (50%-75%) decrease in the number of cortico-STN synapses [108].

### SP promotes and enhances synchronization

We examined how the heterogeneous networks evolved with *hSP-only*. hSP has been used to automatically generate networks that evolve to attain a target activity level [49, 81, 82]. We generated networks using BvOSP and stochastic SP separately from completely unconnected neurons that built contacts in order to reach a given target firing rate, *f*_T_. The neurons with a natural firing rate below *f*_T_ could build new incoming contacts while the ones with higher rates could not. Consequently, a sufficiently large *f*_T_ led to a sufficiently large average node degree that enabled the network to settle in a synchronized state while a lower target allowed only for desynchronized or partially synchronized states. Our stochastic SP method, thus, offers a simpler way to generate networks with the desired level of activity and synchrony.

Next, we developed networks from unconnected neurons using *STDP+SP* (*P*_w_ > 0) and *STDP+hSP* (*P*_w_ = 0) and compared their transition to synchronized states as a function of network density with networks generated using *hSP-only* and the random networks with *STDP-only*. The network developed from scratch using *hSP-only* required the highest average node degree to achieve synchrony, while the network with *STDP-only* could get synchronized with a much smaller average node degree. The smallest average node degree was required by the networks with *STDP+SP* and *STDP+hSP* to reach a synchronized state.

We studied the synchronization dynamics of heterogeneous networks with a combination of STDP and SP. To this end, we considered random networks in synchronized states with *STDP-only* and applied structural updates (SP iterations), as outlined in the *‘Parameters and implementation’* section. As the network progressed through SP iterations, the neurons became increasingly more identical by adding or losing contacts as their firing rates converged to a single target rate with both SP methods (stochastic and BvOSP) regardless of weight-dependent pruning, making way for enhanced network synchronization for any value of the probability of weight-dependent pruning, i.e., *P*_w_ ∈ [0, 1]. Nevertheless, *P*_w_ determined the final average node degree for a given *f*_T_ such that a higher *P*_w_ resulted in a sparser network.

### SP optimizes the network structure by preserving the synaptic contacts that support synchronization

The average synaptic weight of both incoming and outgoing contacts displayed a strong dependence on their natural firing rate in the presence of STDP. Particularly, the average incoming weight decreased with an increase in the natural firing rate, while the average outgoing weight increased since the contacts directed from neurons with a higher natural firing rate to those with a lower rate got potentiated on average while the others got depressed. As the initially random network in the synchronized state underwent structural updates in the presence of weight-dependent pruning, the potentiated contacts survived for longer times, while the depressed ones eventually got pruned. As a consequence, the structure of the network in the presence of SP underwent a reorganization governed by the natural firing rates of the neurons.

With the stochastic SP method, the above factors resulted in a strong relationship between the natural firing rate of the neurons and their in- and out- degrees, such that the neurons with higher (lower) natural firing rates had more (fewer) outgoing and fewer (more) incoming contacts, suggesting that effectively the faster neurons (those projecting densely onto others) drove the slower ones (those receiving more inputs). This finding matches with the experimental observation that in the suprachiasmatic nucleus in the brain, the neurons in the core project heavily onto the neurons in its shell while receiving sparse inputs from the shell [109, 110] and drive synchrony by leading the phase and entraining the neurons in the shell [111]. With BvOSP (*P*_w_ > 0), the dependence of out-degree on the natural firing rate of the neurons in a steady state was different than that with the stochastic SP model since BvOSP identically controlled both in- and out-degrees via firing rate-dependent sprouting of both dendritic and axonal elements, such that the neurons with a natural firing rate close to the target formed fewer dendritic and axonal elements, while our stochastic SP did not directly constrain the out-degree. With BvOSP, the out-degree first increased with an increase in the natural firing rate, reached a maximum, and dropped to a small value near *f*_T_, unlike with stochastic SP where the out-degree monotonously increased with the natural firing rate. With *P*_w_ = 0, both methods produced no degree-frequency correlation.

The removal of weak contacts due to weight-dependent pruning could be viewed as a possible way of optimizing the network structure for synchrony. In a theoretical study, robust synchronization was suggested to benefit from the removal of links [107]. We previously observed such optimization of network structure in a study with networks of oscillators, where the networks that evolved with a combination of STDP and SP got synchronized for significantly smaller average degrees compared to random networks with STDP alone [53].

The degree-frequency correlation above offers insight into the co-evolution of neuronal activity and structure as the activity level of neurons governs the degrees of the network, and the degrees, in turn, alter the level of activity of the neurons. Degree-frequency correlation has been observed in adaptive networks of oscillators where the correlation emerged as a result of either preserving contacts between out-of-phase oscillators while rewiring those between more in-phase ones [50] or due to the dependence of the synaptic weight on the frequency difference between the connected oscillators, accompanied by weight-dependent pruning [53], similar to the present study.

### A mixture of assortativity emerges in synchronized networks with *STDP+SP*

In a steady synchronized state of a network with *STDP+SP* (*P*_w_ ≠ 0), more neurons that remained connected had similar natural firing rates. Since the in- and out-degrees of a neuron depended oppositely on its natural firing rate for a sufficiently large diversity in natural firing rates, the networks portrayed a mixture of degree assortativity by becoming in- and out-assortative while remaining disassortative with respect to the opposite degree types of the connected neurons. The random networks we considered were found to be non-assortative, i.e., Pearson correlation coefficient = 0, similar to previously reported findings [56, 112]. For a weighted graph, the weighted degree should be used to calculate the assortativity [91, 113]; however, for consistency and simplicity, we reported only node degree assortativity. Large-scale networks in the human cerebral cortex were found to be assortative using undirected graph analysis, and the same networks when randomized became non-assortative [114]. Assortative neural networks are also suggested to be more robust in information storage against noise [115]. On the one hand, the assortative nature makes a network robust against node failure or targeted attack and increases the speed of the information transfer [56, 87, 88]. On the other hand, it makes networks more unstable or vulnerable to external perturbation [116].

### Desynchronized networks with combinations of weight and structural plasticity

Next, we studied the transition of the network with *STDP+SP* to a desynchronized state for various values of the weight-dependent pruning probability, *P*_w_ ∈ [0, 1]. The average node degree decreased with an increase in *P*_w_ when the homeostatic probability of addition and pruning, *P*_*h*_, remained fixed. A comparison of the node degrees in the steady synchronized and desynchronized states revealed that depending on the ratio of *P*_w_ to *P*_*h*_, the network in the two states could have significantly different average node degrees. Particularly, comparable values of *P*_w_ and *P*_*h*_ made the desynchronized network considerably denser than the synchronized network, while the larger ratios, *P*_w_/*P*_*h*_, could make the synchronized network much denser than the desynchronized one. In our previous study with simple phase oscillator networks, where the weight-dependent pruning was 100 times faster than homeostatic SP, the desynchronized networks with STDP+SP ended up being considerably sparser than the synchronized network.

In summary, we found that the network structure is strongly related to its dynamical state, and the networks can spontaneously enter pathological or physiological states depending on initial conditions. Future studies should also take into account the impact of stimulation on both abnormal and physiological connectivity, e.g., to improve restoration of segregated activity and related connectivity. By the same token, plasticity mechanisms are crucial for physiological mechanisms. For instance, studies on networks of the interaction of organ systems have demonstrated a similar phenomenon where the network structure is correlated with specific physiologic function such that a change in the interaction network structure is associated with a change in the function [117–119]. Furthermore, the brain wave network interaction and its plasticity are shown to be crucial in creating physiological states and distinct interaction patterns [119, 120]. Accordingly, beyond the specifically desynchronization-oriented approach mentioned above, our model aims to provide a testbed for further development of stimulation methods that cause long-lasting stimulation effects by reshaping connectivity and related neuronal activity in the presence of different plasticity mechanisms.

### SP makes synchronized networks more robust against desynchronizing stimulation

The structure of a network in a synchronized state is critical in determining its robustness against external perturbation. Thus, studies of network structure properties hold importance in the design of therapeutic stimulation methods that have the potential to induce long-lasting relief from the severe effects of pathological synchrony in particular brain areas in certain neurological disorders. Some of the theoretically developed versions of CR stimulation successfully induced long-lasting desynchronizing and/or therapeutic effects in pre-clinical and clinical studies in PD and clinical studies in tinnitus patients as well as in animal models of epilepsy and binge alcohol drinking [71–73, 75–78, 121–123]. RR stimulation [21, 22] and other multichannel stimulation techniques [124, 125] were theoretically developed to induce long-term desynchronizing effects. We here introduced UMRS which shares the temporal randomness of RR stimulation, combined with the spatially fixed multi-site setup of several previous multichannel stimulation techniques [124, 125]. It reliably induced long-term desynchronization in our model networks. While the assortative nature of the networks that evolve with the combination of STDP and SP suggests that they should be more vulnerable to stimulation, the optimization of the network structure for stronger synchrony due to SP suggests that such networks should require stronger stimulation than the random networks with similar average node degrees. The latter outweighed the former in our study since we found that the random network with a similar average node degree as the one that evolved with SP required significantly lower stimulation to get desynchronized. A variety of different desynchronizing stimulation techniques were computationally developed to achieve optimal long-term desynchronization for specific conditions and constraints [21, 22, 24, 33, 126–128]. Analogously, given the slow SP time scale, desynchronizing stimulation techniques can be further developed using network models with STDP and SP with acceptable computation time requirements, hence enabling thorough computational analyses (see, e.g., Refs. [47, 53, 129]). Minimal network models, as used in this study, may enable predictions for the numerical study of more complex, biologically more realistic networks [52, 130–134]. Accordingly, beyond the specifically desynchronization-oriented approach mentioned above, our model aims to provide a testbed for further development of stimulation methods that cause long-lasting stimulation effects by reshaping connectivity and related neuronal activity in the presence of different plasticity mechanisms.

## Supporting information

S1 Text Simulation results using BvOSP in place of stochastic SP model. (PDF)
