# Supplementary material for: Synaptic reorganization of synchronized neuronal networks with synaptic weight and structural plasticity
Source: PLoS Comput Biol. 2024 Jul 9;20(7):e1012261. doi: 10.1371/journal.pcbi.1012261 (PMC11259284; doi:10.1371/journal.pcbi.1012261)
Supplement: S1 Text — (PDF) [file pcbi.1012261.s001.pdf]

Supplementary to Synaptic reorganization of synchronized neuronal networks with synaptic weight and structural plasticity

Butz and van Ooyen SP model (BvOSP)

In this section, we outline a simplified version of the SP model introduced in [1] (BvOSP) and implemented in several studies [1–3] to reproduce experimentally observed network reorganization after lesion and to develop network structure from scratch. In each structural update (i.e., SP iteration) using this method, the synaptic elements, both axonal boutons and dendritic spines, of a neuron are created and deleted based on its average activity level. The available complementary synaptic elements of different neurons are paired together, i.e., an axonal bouton of one neuron is combined with a dendritic spine of the other, to form synaptic contacts. The remaining elements are made available for contact formation in the following iteration of the structural update. In the original model [1], a neuron's average activity is depicted by its intracellular calcium concentration in accordance with experimental findings. However, the time-averaged firing rate could also represent the average neuronal activity since the calcium concentration and the firing rate are directly proportional [4]. We use the time-averaged firing rate here, consistent with the stochastic SP model in the main text.

We simplified the BvOSP model as follows. The birth and death of synaptic elements of neuron i depend on its firing rate, f_i, and the homeostatic set-point firing rate (target firing rate), f_{T}. The synaptic elements are created when the activity of a neuron falls below f_{T} and are deleted if it goes above f_{T}. We assume a linear dependence of the change in the number of synaptic elements on f_i.

$$\frac{dz_i}{dt} = \nu_{\mathrm{sp}} \left(1 - \frac{f_i}{f_{\mathrm{T}}}\right), \tag{1}$$

where z represents both axonal and dendritic elements, i.e., $z \in \{Ax, Dn\}$. ν_{sp} is the sprouting rate of the synaptic elements. We assume the same sprouting rate for both axonal and dendritic elements. At any given time, a synaptic element may be either bounded (as part of an existing synaptic contact) or vacant (available to form contacts). The detailed algorithm of addition and pruning of synaptic contacts is presented in Refs. [1–3]. Briefly, dz_i is calculated for each neuron until the following SP iteration and rounded to the smaller integer number. If $dz_i > 0$, that many elements are created and added to the pool of vacant elements, while if $dz_i < 0$, that many previously existing elements of neuron i are randomly selected and deleted, irrespective of their state (bounded or vacant). If the deleted element belonged to a synaptic contact,

the corresponding synaptic contact is pruned and the counterpart element becomes vacant and available for contact formation. The number of vacant elements decays spontaneously with a time constant, τ^{vac} [1],

$$\frac{dz_i^{\text{vac}}}{dt} = -\frac{z_i^{\text{vac}}}{\tau^{\text{vac}}}.$$

The addition process is carried out by simply collecting the vacant synaptic elements and randomly assigning them to their counterparts, which causes a given postsynaptic (presynaptic) neuron to be more likely to connect to a presynaptic (postsynaptic) neuron with a larger number of vacant axonal (dendritic) elements [1]. The contacts are formed if the distance-dependent probability permits. We use the same exponentially decaying distance dependence here as for the stochastic SP in the main text for the formation of a contact. Besides the conceptual difference, a key difference between BvOSP and the stochastic SP model is that BvOSP determines the number of outgoing contacts from presynaptic neurons based on their activity level via activity-dependent axonal bouton generation, while the stochastic SP model does not control the number of outgoing contacts.

BvOSP models only the homeostatic SP, meaning it does not include synaptic weight-dependent pruning. We introduce the weight-dependent pruning of synaptic contacts, in which both axonal and dendritic elements engaged in that contact get deleted, to make it comparable to the stochastic SP model. A maximum of $\mathcal{M}$ contacts can be built between a pair of pre- and post-synaptic partners, i.e., $k = 1, 2, .., \mathcal{M}$, and each contact between a pair can have a different weight at any given time. The change in the synaptic weights is governed by Eq. 7 in the main text. Only the contacts that become weak enough to get eliminated are pruned with the weight-dependent pruning probability (Eq.13 in the main text), while the others between the same pair remain intact.

Here we replace the homeostatic addition and pruning of our stochastic SP model introduced in the main text with BvOSP to compare the emerging network states and structure resulting from the two distinct models. As done in the main text using the stochastic SP model, we consider networks with

1. homeostatic SP (hSP) alone, i.e., without weight-dependent pruning ($P_{\text{w}} = 0$) and without STDP, labeled as *hSP-only*;
2. a combination of STDP and hSP, i.e., without weight-dependent pruning ($P_{\text{w}} = 0$), labeled as *STDP+hSP*;
3. a combination of STDP and SP that includes hSP and weight-dependent pruning ($P_{\text{w}} \neq 0$), labeled as *STDP+SP*,

The BvOSP model is implemented as follows. The network is either initialized with random graph

connectivity or completely unconnected. dz_i is calculated at every time-step and allowed to change over the window of duration ΔT_{sp} during which no SP updates are executed and the network is allowed to settle in a steady state either with or without STDP, determined by Eq. 19 in the main text. The sprouting rate, $\nu_{\text{sp}} = 10^{-5}\text{ms}^{-1}$ and $\tau^{\text{vac}} = 10\Delta T_{\text{sp}}$, comparable to previous studies [1–4]. The addition of a synaptic contact from a neuron j to i increases the adjacency matrix element $A_{i,j}$ by 1 while the pruning of that reduces $A_{i,j}$ by 1 with the upper limit at $\mathcal{M} = 10$ and lower at 0. The newly added contacts are given a random weight between 0 and 0.2 unless stated otherwise.

Results

Networks with *hSP-only*

With BvOSP, we obtain similar results as with the stochastic SP method in the main text. First, we develop networks from completely unconnected neurons employing *hSP-only* with a given target firing rate, f_{T}. We consider $f_{\text{T}} = 3, 4,$ and 5 Hz. The newly added contacts are given a random weight between 0 and 1. The network-averaged firing rate increases with SP iterations as neurons develop synaptic contacts with other neurons to achieve f_{T}, increasing the average NDD as shown in Fig A(a,c). Accordingly, a lower f_{T} leads to a lower average NDD in the steady state, which may prevent the network from getting synchronized due to an insufficient number of contacts. Thus, a network with low f_{T} settles in a desynchronized state, while those with larger values of f_{T} may get partially or fully synchronized, as shown for $f_{\text{T}} = 4$ and 5 Hz, respectively, in Fig A(b). A lower CV of firing rates accompanies a higher order parameter [Fig A(d)].

Neurons with a natural firing rate lower than f_{T} develop more dendritic and axonal elements, leading to larger values of in- and out-NDDs, respectively. We observe an identical decrease of both in- and out-NDDs with an increase in the natural firing rates of the neurons in Fig B(a,b,c) for all values of f_{T} considered since the number of axonal and dendritic elements follow the same dependence on f_i and f_{T} (Eq. 1). The neurons with a natural firing rate above f_{T} may remain unconnected, e.g., for $f_{\text{T}} = 3$ and 4 Hz, as they may not develop any synaptic elements. Consequently, the joint probability distributions in Fig B(d,e,f) show a strong positive correlation between in- and out-NDDs.

Networks with combinations of structural and weight plasticity

We consider synchronized random networks with *STDP-only* and apply structural updates using *STDP+SP* ($P_{\text{w}} = 0.01$), as outlined in the *"Parameters and implementation"* section in the main text, to investigate the effect of SP on such networks. We consider three random networks with initial average NDDs, $\beta_0 = 0.075, 0.11,$

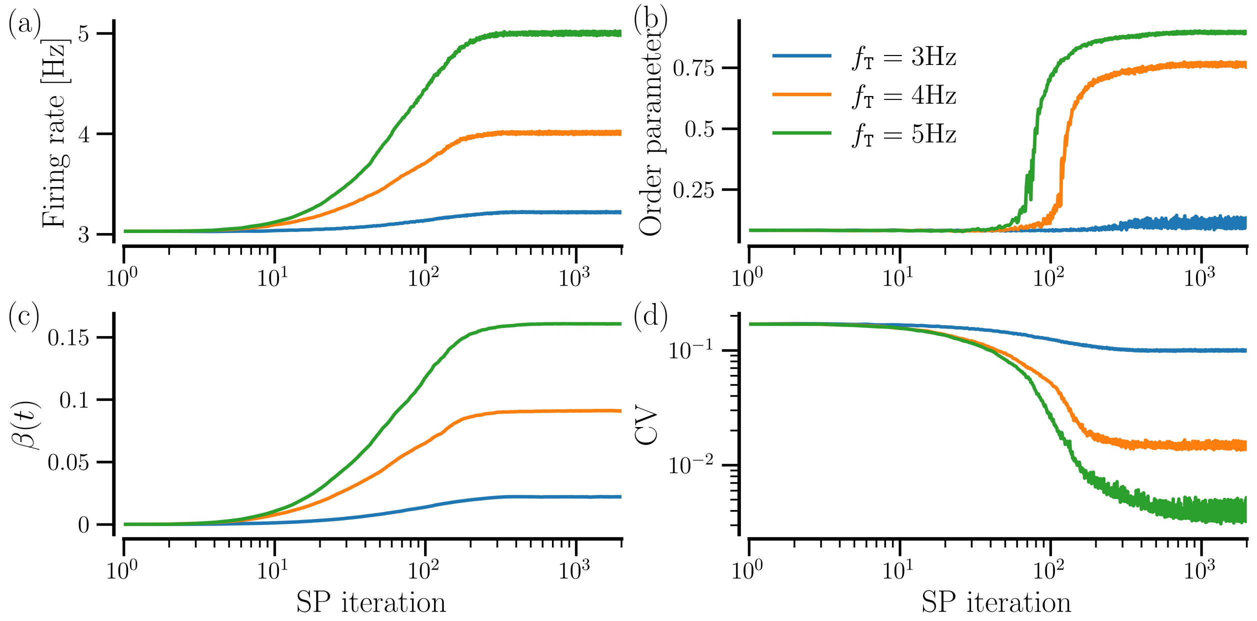

Fig A. Time evolution of the state measures of networks developed with *hSP-only* starting from unconnected neurons. (a) shows the network-averaged firing rate, (b) the order parameter, (c) the network-averaged NDD, and (d) the CV of firing rates of the neurons. Parameter: sprouting rate, $\nu_{\text{sp}} = 10^{-5}$ ms^{-1}.

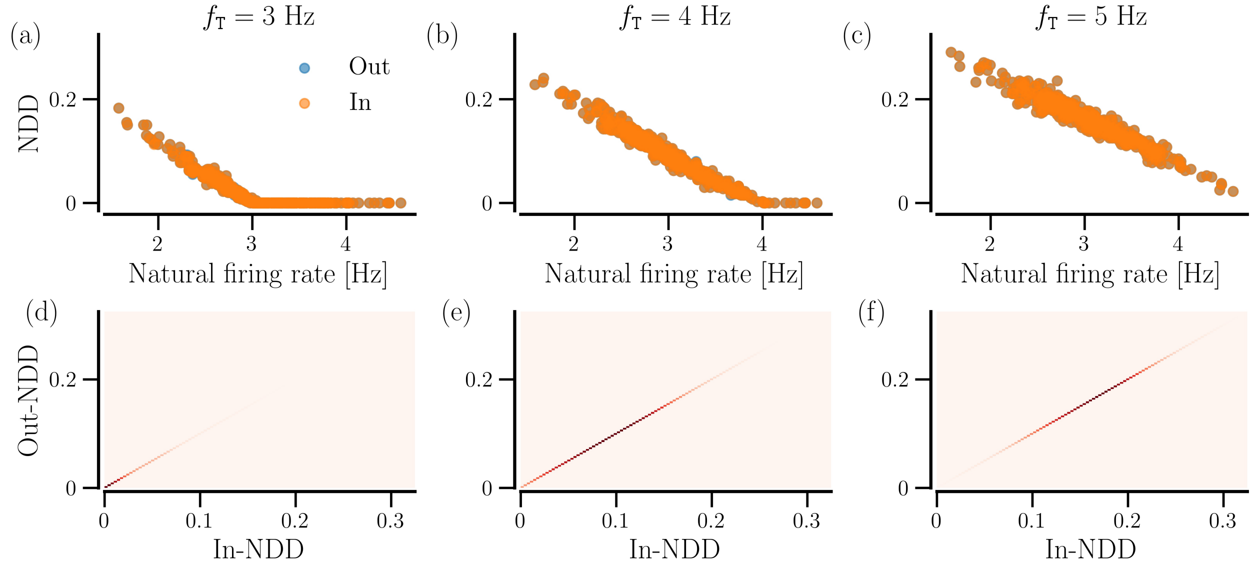

Fig B. Dependence of in- and out-NDDs on the natural firing rate of the neurons in a steady state and their joint probability distribution, when unconnected neurons form networks using *hSP-only*. From left to right, the top and bottom panels correspond to the target firing rates, f_T, specified at the top. Top panels show the in- and out-NDDs as functions of the natural firing rate of the neurons and the bottom panels show the joint probability distribution of in- and out- NDDs, P(in-NDD,out-NDD). Parameter: sprouting rate, $\nu_{\text{sp}} = 10^{-5}$ ms^{-1}.

and 0.2. Fig C shows the evolution of the dynamical measures of the network as it progresses through SP iterations. The network-averaged firing rate evolves towards f_T for all initial average NDDs as shown in Fig C(a). Since the target firing rate and probabilities of addition and pruning are the same for all three

initial NDDs, the networks settle with the same average NDD in the steady state, regardless of the initial value, as shown in Fig C(c). Importantly, the networks may become more strongly synchronized as indicated by the increase in the order parameter in Fig C(b) and the decrease in the firing rate CV in Fig C(d).

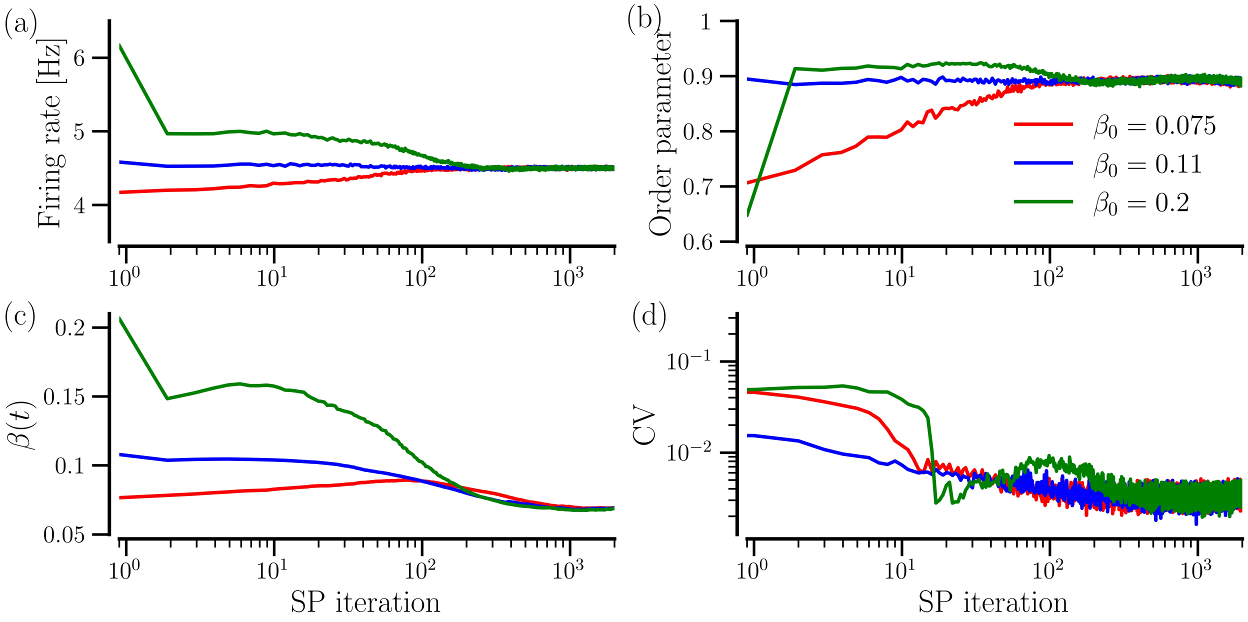

Fig C. Evolution of network-averaged measures with SP iterations. Colors correspond to the three initial values of average NDD (see legend). (a) shows the network-averaged firing rate, (b) the order parameter, (c) the network-averaged NDD, and (d) the CV of firing rates of the neurons. The parameters are: $f_{\mathrm{T}} = 4.5$ Hz, $P_{\mathrm{w}} = 0.01$, $\langle W \rangle(0) = 0.8$.

We further examine the impact of taking a synchronized random network with *STDP-only* through structural updates using either *STDP+hSP* or *STDP+SP* ($P_{\mathrm{w}} = 0.01$). Fig D(a) shows that with both $P_{\mathrm{w}} = 0.01$ (*STDP+SP*) and $P_{\mathrm{w}} = 0$ (*STDP+hSP*) the network reaches the given target rate. Fig D(b and d) show that network synchronization gets equally enhanced for both *STDP+hSP* or *STDP+SP* as the network evolves, compared to the original synchronized random network with *STDP-only*, as indicated by the increase and decrease in the order parameters and firing rate CVs, respectively. However, the average NDD is considerably lower in the presence of weight-dependent pruning (*STDP+SP*) owing to the elimination of weaker contacts [Fig D(c)], pointing to the optimization of network structure in the synchronized state, allowing for strong synchrony with sparser networks.

The average incoming and outgoing synaptic weights of neurons depend strongly on their natural firing rate in the presence of STDP in a synchronized state, such that the average weight of incoming contacts of neurons with a low (high) natural firing rate is high (low), while that of outgoing contacts is low (high) as shown in Fig E(a). Accordingly, the distribution of the synaptic weight of contacts shows peaks at 0 and 1 with *STDP-only* in Fig E(b). Similar to that for the stochastic SP method in the main text, we observe

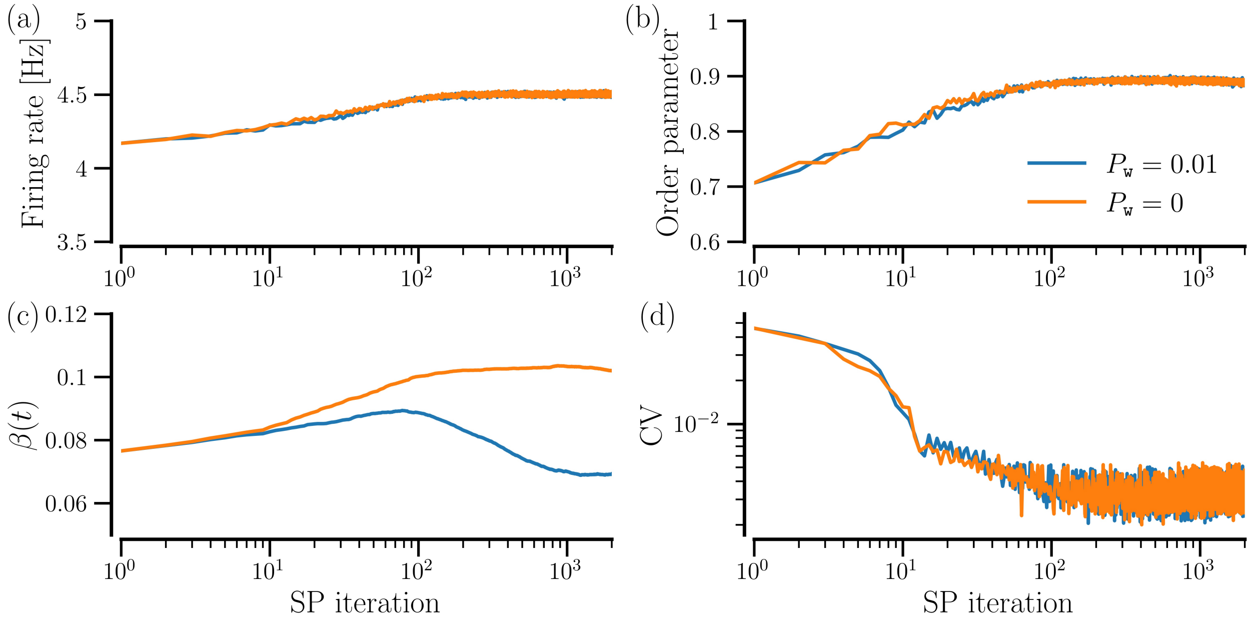

Fig D. Evolution of network-averaged measures with SP iterations with and without weight-dependent pruning, $P_w = 0.01$ (*STDP+SP*) and $P_w = 0$ (*STDP+hSP*), respectively. (a) shows the network-averaged firing rate, (b) the order parameter, (c) the network-averaged NDD, and (d) the CV of firing rates of the neurons. Parameters: $\beta_0 = 0.075$ and initial weight $\langle W_0 \rangle = 0.8$.

using BvOSP that the peak at 0 disappears in the presence of weight-dependent pruning but not for $P_w = 0$. The number of strong contacts in the steady states for all three cases remains high.

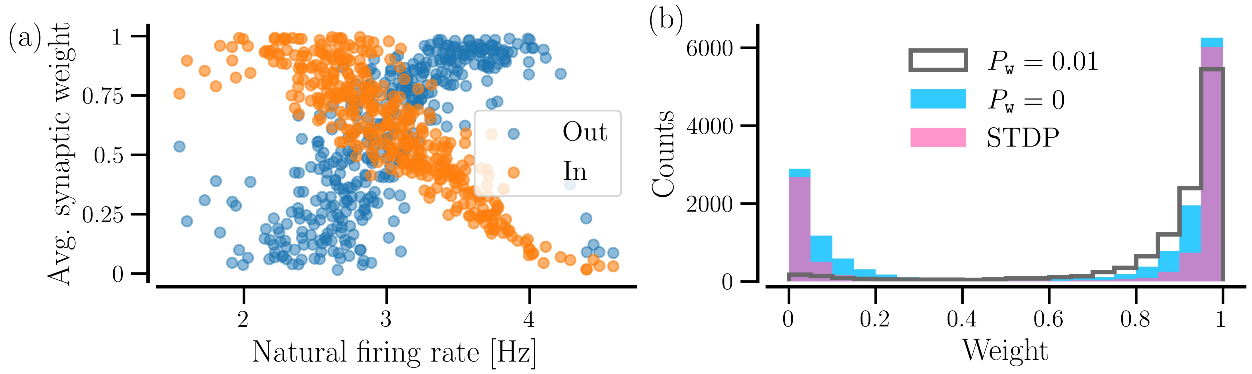

Fig E. Dependence of average incoming and outgoing synaptic weight on the natural firing rate of neurons for a random network with *STDP-only* (a) and the distributions of the synaptic weight for random network with *STDP-only*, *STDP+hSP* ($P_w = 0$), and *STDP+SP* ($P_w = 0.01$) (b). Other parameters are the same as for Fig D.

Eq. 1 dictates that the lower the natural firing rate compared to the target rate, the higher the numbers of axonal and dendritic elements of the neuron. Axonal and dendritic elements are respectively responsible for outgoing and incoming synaptic contacts of a given neuron. With *STDP+SP* ($P_w = 0.01$), out-NDD becomes low and in-NDD remains high for neurons with low natural firing rates because of the pruning of their weaker contacts in Fig F(a). The out-NDD increases and the in-NDD decreases with an increase in the

natural firing rate, following the synaptic weight dependence on the natural firing rate of the neurons shown in Fig E. With *STDP+hSP* ($P_{\mathrm{w}} = 0$), both in- and out-NDDs are high for neurons with low natural firing rates and show a minor drop with an increase in the natural firing rate of the neurons in Fig F(b) due to the absence of preferred pruning of weak contacts. Neurons with a natural firing rate close to the target rate have few to no synaptic elements. A small number of axonal elements leads to a low out-NDD, while that of dendritic elements results in a low in-NDD for both $P_{\mathrm{w}} = 0.01$ and $P_{\mathrm{w}} = 0$. Thus, we observe higher natural firing rate neurons with minimal in- and out-NDD in Fig F(a,b).

Fig F(c,d) show the joint probability distributions of the in- and out- NDDs of individual neurons for $P_{\mathrm{w}} = 0.01$ and $P_{\mathrm{w}} = 0$, respectively. For $P_{\mathrm{w}} = 0.01$, neurons with a high in-NDD have a low out-NDD, and most of the neurons with low in-NDD have a high out-NDD, resulting in a negative correlation between the two. For $P_{\mathrm{w}} = 0$, on the contrary, the in- and out- NDDs depend similarly on the natural firing rate of the neurons, and thus, assume similar values leading to a positive correlation between the two.

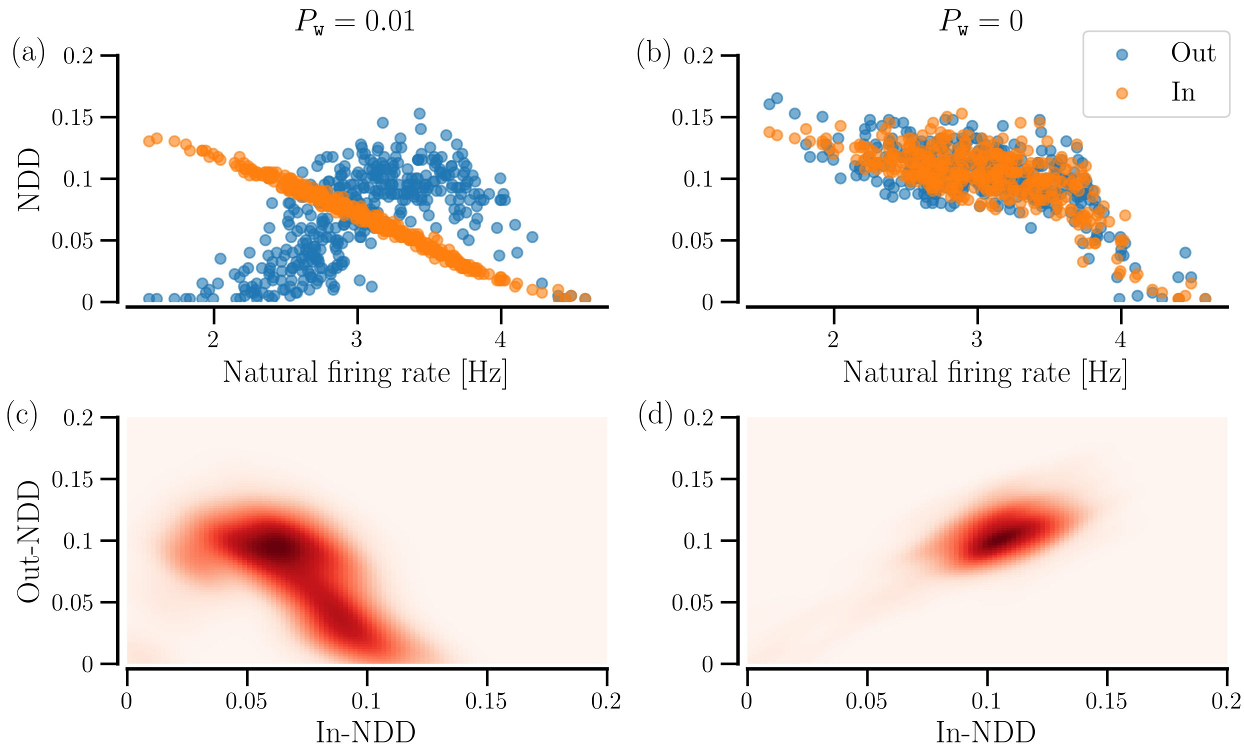

Fig F. Dependence of in- and out- NDD on the natural firing rates of the neurons for *STDP+hSP* ($P_{\mathrm{w}} = 0$), and *STDP+SP* ($P_{\mathrm{w}} = 0.01$) and their joint probability distribution, P(in-NDD,out-NDD) in the steady synchronized states. (a) and (b) show the dependence of the in- and out- NDD on the natural firing rates of the neurons for $P_{\mathrm{w}} = 0.01$ and $P_{\mathrm{w}} = 0$, respectively. (c) and (d) show the corresponding joint probability. Other parameters are the same as for Fig D.

Fig G compares NDD distributions in steady synchronized states for $P_{\mathrm{w}} = 0$ and $P_{\mathrm{w}} = 0.01$. For $P_{\mathrm{w}} = 0$, both in- and out-NDD distributions have a similar shape and possess the same mode. The accumulation of

weaker contacts due to the absence of their preferential pruning results in a shift of the in- and out-NDDs to higher values compared to the initial distributions. When $P_{\mathrm{w}} = 0.01$, both in- and out-NDD distributions are shifted towards smaller values and have smaller peaks compared to the initial because of the pruning of weaker contacts, indicating that more neurons end up with smaller NDD and fewer than initial neurons have high NDD.

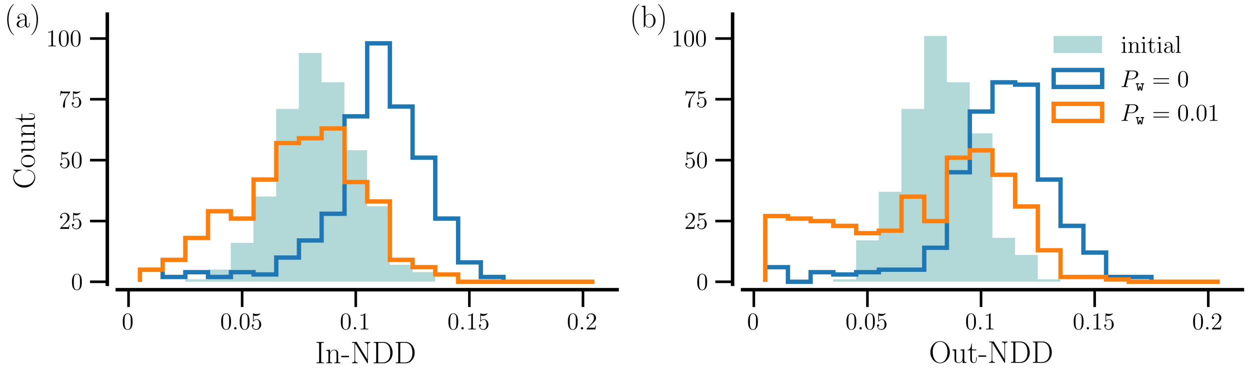

Fig G. Marginal distributions of in- and out- NDDs for the network in the steady synchronized states with *STDP+hSP* ($P_{\mathrm{w}} = 0$) and *STDP+SP* ($P_{\mathrm{w}} = 0.01$). (a) shows the in-NDD and (b) shows the out-NDD. Both show the distributions for *STDP+hSP* ($P_{\mathrm{w}} = 0$) and *STDP+SP* ($P_{\mathrm{w}} = 0.01$). The blue-filled histogram shows initial degree distributions. Other parameters are the same as for Fig D.

Statistics of synaptic contacts between a pair of pre- and post-synaptic partners

We restrict the number of contacts from a presynaptic neuron to each of its postsynaptic partners with BvOSP to a maximum of 10, as previously suggested [5, 6]. Fig H shows the number of contacts (counts) established from a presynaptic neuron to each of its postsynaptic partners in three cases: networks developed from unconnected neurons using *hSP-only* for given target firing rates, f_{T}, synchronized (initially) random networks with different initial NDDs, β_0, that evolved with *STDP+SP* ($P_{\mathrm{w}} = 0.01$), and those that evolved with either $P_{\mathrm{w}} = 0.01$ or $P_{\mathrm{w}} = 0$. The counts remain much smaller than 10, and thus, may be unaffected by the imposed maximum, $\mathcal{M}$, as previously observed [6].

All panels in Fig H show the highest peak at 0 contacts, indicating that most pairs remain unconnected, consistent with the small average NDDs. The counts decrease with the increase in the number of contacts, implying that more pairs tend to develop a smaller number of contacts. For networks developed from scratch with *hSP-only* for given f_{T}, Fig H(a) shows that for a higher f_{T}, the counts for non-zeros contacts tend to be higher, since the network's average NDD increases with an increase in the target [cf. Fig A(c)]. Fig H(b) shows that in synchronized states of networks with *STDP+SP* ($P_{\mathrm{w}} = 0.01$) the counts for all numbers of contacts are similar, since the networks settle with the same steady-state average NDD [Fig C(c)], regardless of the initial average NDD. Consistent with the larger average NDD observed for $P_{\mathrm{w}} = 0$ than $P_{\mathrm{w}} = 0.01$,

Fig H(c) shows that the peaks at the non-zero numbers of contacts are higher for $P_w = 0$.

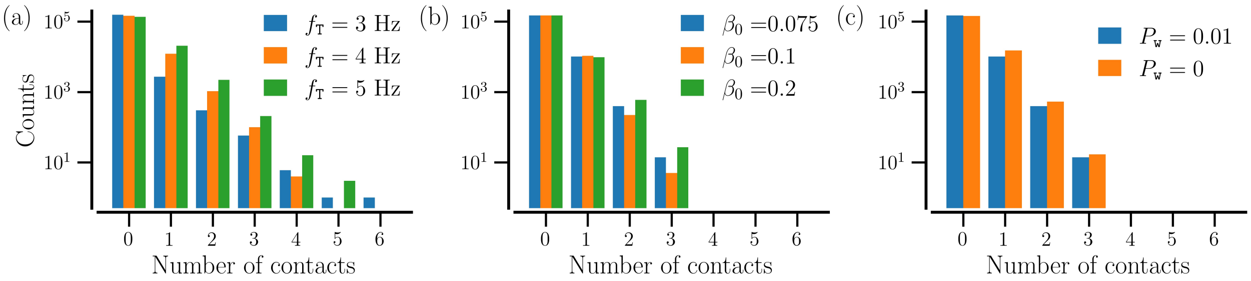

Fig H. The numbers (counts) of the pairs of pre-and post-synaptic partners that possess a given number of contacts in the steady state for networks developed from unconnected neurons using *hSP-only* for given target firing rates, f_T (a), synchronized (initially) random networks with different initial NDDs, β_0, that evolved with *STDP+SP* ($P_w = 0.01$) (b), and those that evolved with either *STDP+SP* ($P_w = 0.01$) or *STDP+hSP* ($P_w = 0$) (c). Parameters for (a), (b), and (c) are the same as for Figs A, C, and D, respectively.

Previous studies that used their specially designed SP methods successfully reproduced experimentally observed bimodal distributions [5–7], where one peak is observed at 0 and the other tends to lie between 3 and 8 [5, 8, 9]. However, we observed a consistently decreasing distribution with the increase in the number of contacts for all the cases we considered: networks generated from completely unconnected neurons using *hSP-only* as well as networks that were initially random but evolved with combinations of STDP and SP or hSP. It is because the multiple contact formation in our study corresponds to the independent synaptic contact formation via a random conversion of potential synaptic contacts to actual synaptic contacts, as previously discussed in Ref. [5]. The distribution of potential synaptic contacts shows a consistent decrease, and so does the actual contact distribution in the present study. A bimodal distribution can be reproduced when the formation of new synaptic contacts between a given pair of neurons is assumed to depend on the number and state of the existing contacts, the specific interaction of neuron activity level, synaptic weight, and rewiring, and/or the presence of other neurons that may compete with the presynaptic partner [5–7, 10]. Here we considered no such dependencies and BvOSP inherently does not take into account the competition between the possible presynaptic partners [1].

Simulation results with networks of 32×32 neurons ($N = 1024$)

In this section, we use the stochastic SP method instead of the BvOSP to show that the results presented in the main text with networks of 400 neurons hold for larger networks. We present an example where we develop networks using *STDP+SP* ($P_w = 0.01$) and *hSP-only* to compare their transition to synchronized states. With *STDP+SP*, the new contacts added at each SP iteration (structural update) are given a random initial weight $\in [0, 0.2]$ and are subjected to STDP allowing those to get potentiated or depressed. Thus, the

neurons can adjust their firing rates by adding and losing contacts besides changing the synaptic weights. With *hSP-only*, the new contacts added at each SP iteration are given a random initial weight $\in [0, 1]$. The target firing rate is set to 4.5 Hz. Since the target firing rate is higher than the natural firing rates of most of the neurons (mean 3 Hz and standard deviation 0.5 Hz), the initial structural updates involve only the addition of new contacts. The network may get synchronized when a sufficiently large number of contacts have developed with progressing SP iterations. This is indicated by an increase of the order parameter in Fig I(a) and a drop of the firing rate CV in Fig I(c). With *hSP-only*, the network may require a larger number of contacts to get synchronized because some of the strong contacts that are added may not support but rather oppose synchronization. However, for the network with *STDP+SP*, the synaptic weight change of the contacts may preclude the need to build more contacts, allowing for synchronization at a significantly earlier stage of network evolution and, thus, for smaller average NDD compared to the network with *hSP-only*. Combining panels (a) and (b) of Fig I and by excluding the SP iteration axis provides the dependencies of the order parameter on β in Fig I(d). Similarly, the dependencies of the firing rate CV on β in Fig I(e) are obtained by combining panels (c) and (b). We add the corresponding curves of the order parameter and the firing rate CV versus average NDD for random networks with *STDP-only* generated for every value of average NDD to compare the transition of networks developed from scratch to random networks with *STDP-only*. Fig I(d) shows that the network with *STDP+SP* transitions to its synchronized state for significantly lower average NDD than networks with *STDP-only* and *hSP-only*. Fig I(b) shows that as the networks pass the onset of synchronization, the increase in average NDD remains smaller for the network with *STDP+SP* compared to that with *hSP-only*. This is further demonstrated in Fig I(d) and I(e), where the curves of order parameter and CV versus β cease to change at smaller values of β for networks with *STDP+SP*. Importantly, Fig I(e) shows that the plasticity of network structure results in synchronized states with much smaller values of the firing rate CV, indicating stronger frequency locking.

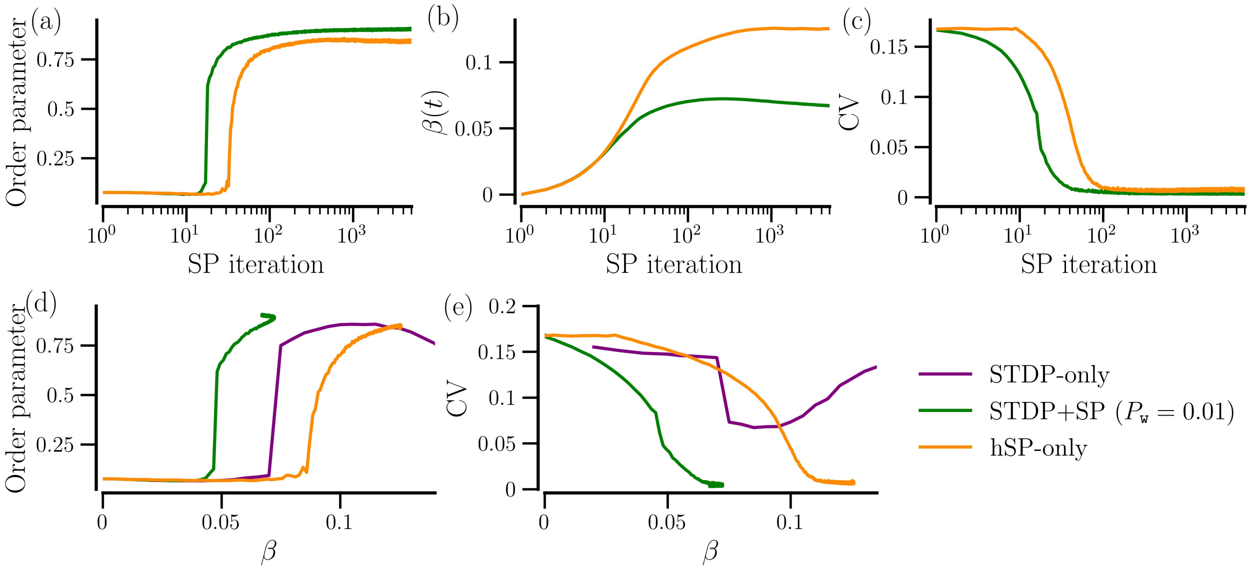

Fig I. Comparison of network evolution in the presence of different plasticity cases. The change in the order parameter, network-averaged NDD, and firing rate CV with SP iterations (a, b, and c, respectively). Order parameter and the CV of the firing rate versus the network-averaged NDD for the three plasticity cases (d and e). The asymmetry parameter, b, of STDP, is set to 1. Parameters: $P_h = 0.01$, $\sigma_f = 0.5$ Hz

References

1. Butz M, van Ooyen A. A simple rule for dendritic spine and axonal bouton formation can account for cortical reorganization after focal retinal lesions. PLoS computational biology. 2013;9(10):e1003259.

2. Butz M, Steenbuck ID, van Ooyen A. Homeostatic structural plasticity increases the efficiency of small-world networks. Frontiers in synaptic neuroscience. 2014;6:7.

3. Diaz-Pier S, Naveau M, Butz-Ostendorf M, Morrison A. Automatic generation of connectivity for large-scale neuronal network models through structural plasticity. Frontiers in neuroanatomy. 2016;10:57.

4. Manos T, Diaz-Pier S, Tass PA. Long-term desynchronization by coordinated reset stimulation in a neural network model with synaptic and structural plasticity. Frontiers in Physiology. 2021;12:716556.

5. Fares T, Stepanyants A. Cooperative synapse formation in the neocortex. Proceedings of the National Academy of Sciences. 2009;106(38):16463–16468.

6. Deger M, Seeholzer A, Gerstner W. Multicontact Co-operativity in Spike-Timing–Dependent Structural Plasticity Stabilizes Networks. Cerebral Cortex. 2018;28(4):1396–1415.

7. Fauth M, Wörgötter F, Tetzlaff C. The formation of multi-synaptic connections by the interaction of synaptic and structural plasticity and their functional consequences. PLoS computational biology. 2015;11(1):e1004031.

8. Markram H, Lübke J, Frotscher M, Roth A, Sakmann B. Physiology and anatomy of synaptic connections between thick tufted pyramidal neurones in the developing rat neocortex. The Journal of physiology. 1997;500(2):409–440.

9. Feldmeyer D, Egger V, Lübke J, Sakmann B. Reliable synaptic connections between pairs of excitatory layer 4 neurones within a single 'barrel'of developing rat somatosensory cortex. The Journal of physiology. 1999;521(1):169–190.

10. Deger M, Helias M, Rotter S, Diesmann M. Spike-timing dependence of structural plasticity explains cooperative synapse formation in the neocortex. PLoS computational biology. 2012;8(9).
